# MetNetGE: interactive views of biological networks and ontologies

**DOI:** 10.1186/1471-2105-11-469

**Published:** 2010-09-17

**Authors:** Ming Jia, Suh-Yeon Choi, Dirk Reiners, Eve S Wurtele, Julie A Dickerson

**Affiliations:** 1 Department of Electrical and Computer Engineering, Iowa State University, Ames, IA, USA; 2 Department of Genetics, Development and Cell Biology, Iowa State University, Ames, IA, USA; 3 Center for Advanced Computer Studies, University of Louisiana, Lafayette, Louisiana, USA; 4 Virtual Reality Applications Center, Iowa State University, Ames, IA, USA

## Abstract

**Background:**

Linking high-throughput experimental data with biological networks is a key step for understanding complex biological systems. Currently, visualization tools for large metabolic networks often result in a dense web of connections that is difficult to interpret biologically. The MetNetGE application organizes and visualizes biological networks in a meaningful way to improve performance and biological interpretability.

**Results:**

MetNetGE is an interactive visualization tool based on the Google Earth platform. MetNetGE features novel visualization techniques for pathway and ontology information display. Instead of simply showing hundreds of pathways in a complex graph, MetNetGE gives an overview of the network using the hierarchical pathway ontology using a novel layout, called the Enhanced Radial Space-Filling (ERSF) approach that allows the network to be summarized compactly. The non-tree edges in the pathway or gene ontology, which represent pathways or genes that belong to multiple categories, are linked using orbital connections in a third dimension. Biologists can easily identify highly activated pathways or gene ontology categories by mapping of summary experiment statistics such as coefficient of variation and overrepresentation values onto the visualization. After identifying such pathways, biologists can focus on the corresponding region to explore detailed pathway structure and experimental data in an aligned 3D tiered layout. In this paper, the use of MetNetGE is illustrated with pathway diagrams and data from *E. coli *and *Arabidopsis*.

**Conclusions:**

MetNetGE is a visualization tool that organizes biological networks according to a hierarchical ontology structure. The ERSF technique assigns attributes in 3D space, such as color, height, and transparency, to any ontological structure. For hierarchical data, the novel ERSF layout enables the user to identify pathways or categories that are differentially regulated in particular experiments. MetNetGE also displays complex biological pathway in an aligned 3D tiered layout for exploration.

## Background

### Biological Pathway Visualization

The availability of high-throughput experimental data provides new possibilities for understanding biological systems and creates new challenges for visualization tools as well. Data in high-throughput experiments can encompass thousands of RNAs, metabolites and/or polypeptides. Mapping such data onto a network that represents the interactions in an organism is essential for biologists to understand how the parts of the system influence each other and to generate data-driven new hypotheses [1-3]. Popular representations of such networks include node-link graphs and the adjacency matrixes. In the node-link graph, nodes represent genes, gene products, metabolites and reactions, and edges represent specific interactions, e.g., transcription, translation, catalysis, and different types of regulation.

A number of publicly accessible pathway databases containing data about genes, gene products, and interactions are available, e.g., BioCyc [2] MetNetDB [3] and KEGG [4]. In order to get better insight from such vast data sets, many graph visualization tools have been developed such as Cytoscape [5] and VisANT [6]. Suderman et al. [7] reviewed 35 such visualization tools and noted key useful features such as generation of good layouts and integration with analysis software. They also pointed out several key drawbacks of those tools. For example, the use of generic layout algorithms often produces network drawings that are very messy with many crossing edges. Furthermore, most tools can not visually represent dynamic information (e.g., gene expression data) at a large-scale, systems-wide level.

Despite the recent emergence of many pathway visualization tools, many tasks are still challenging. One important challenge is generating a meaningful visualization of the whole network. Unfortunately, generic layout algorithms such as the *yFiles Organic*, force-directed layout style [8] in Cytoscape often result in the notorious 'hair-ball' view for large networks (Additional file 1, Figure 1). Unstructured views that do not take biological information into account give the user very little information about the structure of the network. Other challenges include showing hierarchical relationships, e.g., pathway and gene ontologies that may reveal hidden functional relationships. These functionalities are crucial for biologists to explore, understand and make connections among experimental data.

**Figure 1:**
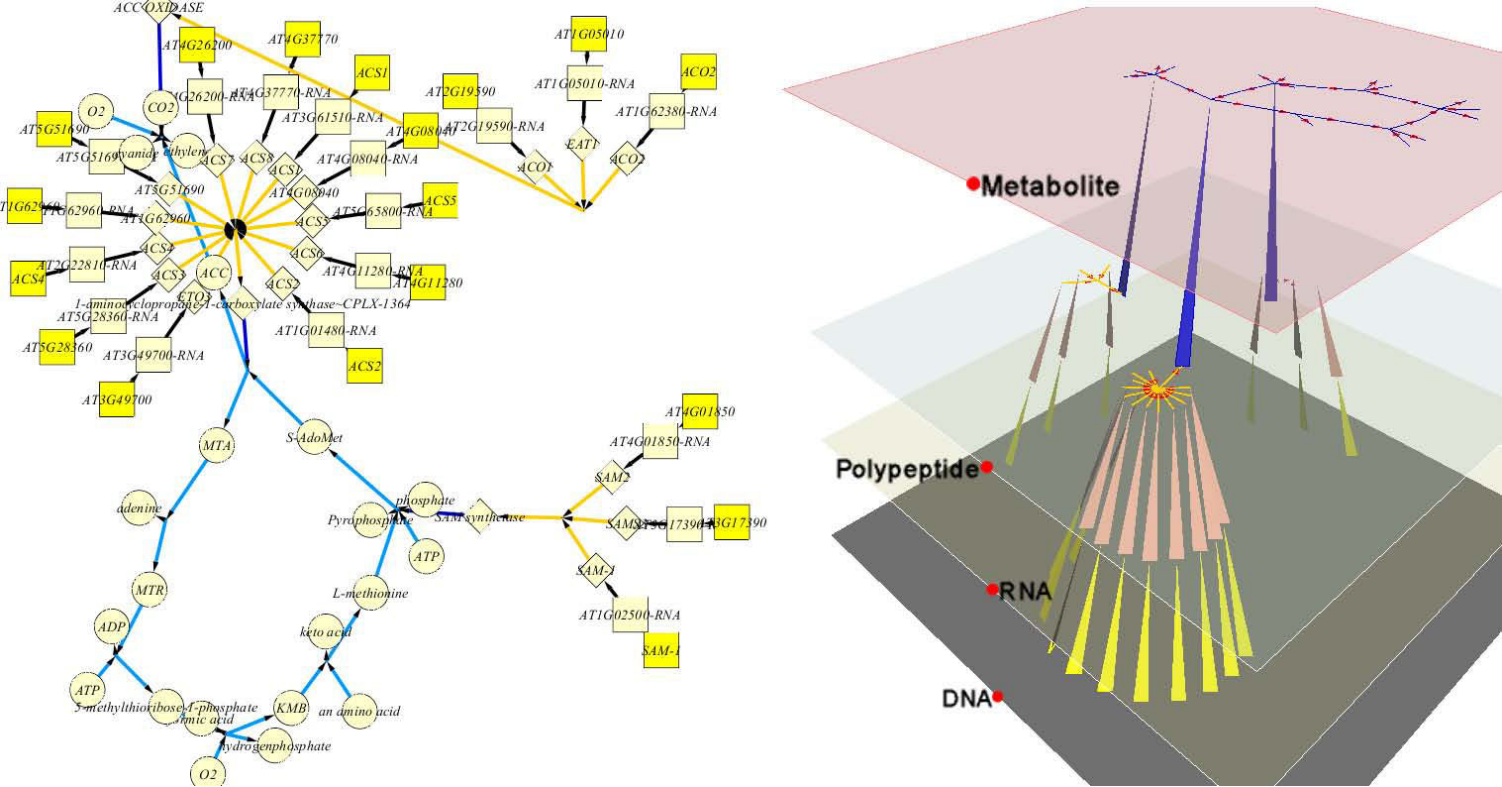
**Visualize one metabolic pathway**. The pathway *ethylene biosynthesis and methionine cycle *is drawn using the yFiles Organic layout style [8] in Cytoscape (left) and 3D tiered layout in MetNetGE (right). The metabolite layer is chosen as the major plane in MetNetGE. The 3D tiered layout shows that the basic metabolic flow is a cycle in the metabolite layer and that three protein complexes catalyze the metabolic reactions (blue edges from the polypeptide layer to the metabolite layer).

3D approaches to display networks may offer methods to display more information [9,10], however most popular bioinformatics tools do not support 3D directly. The use of stacked 2D layouts was introduced in [11], where similar pathways across several species are compared. This representation is very effective at highlighting small differences between two species; however it cannot be directly applied to pathway diagrams since adjoining pathways do not have common structures.

Arena3D [12] puts nodes into different layers to reveal interactions between node types. BioCichlid [13] divides protein and genes into separate layers in 3D to look at genetic regulation. These works show the promise of using an extra dimension where the network complexity is reduced by separating the whole graph into several 2D planes. However, since BioCichlid and Arena3D compute separate layouts for each layer, edges between layers are often cluttered and difficult to follow. MetNetGE uses a 3D tiered layout for each individual pathway where the pathway algorithm aligns the layouts in each plane based on the most important plane. This helps the basic pathway structure stand out and create a clearer and easily understandable drawing.

### Requirements for Visualizing Biological Ontologies

In addition to being able to visualize a network organized in a meaningful manner, biologists need an overview of broader functional categories and network performance under different experimental conditions, e.g., to be able to ask questions such as whether degradation pathways have many highly expressed genes, or which biological process categories are overrepresented in the data. The remainder of this paper will use graph terminology to describe the ontology and visualization techniques. Thus, the term "tree" means the data structure, "leaf" node means the node in the tree structure that does not have any children, and "non-leaf" node means the node with at least one child, and is not related to the organism of a plant.

Controlled vocabularies are graph-theoretical structures consisting of terms (which form the nodes of each corresponding graph) linked together by means of edges called relations [14]. Structured hierarchical ontologies such as the Pathway Ontology (PO) and Gene Ontology (GO) impose biological relationships on the network which can aid in interpretation. For example, the PO can be used for analysis tasks such as identifying pathways that belong to multiple biological categories. Pathways that belong to multiple categories are ontological terms with multiple inheritance, which is not easily presented by most visualization tools [15]. The GO and PO can be combined with experimental data as well to see if any categories or terms are statistically overrepresented by mapping the experimental data and its aggregated values onto the ontology of interest. After mapping, biologists need to evaluate which categories are significantly different among the experimental conditions and establish how those categories are related in the hierarchy. Based on the task types that biologists typically perform, the basic requirements for biological ontology visualization are to:

• View the whole ontology on a single screen to gain a global feeling for the data and the main hierarchical structure.

• View ontology details by navigation and/or interaction (zoom, pan, rotation).

• Map attributes on the ontology so that they are easily visible.

• Clearly show non-tree connections.

Normally, one single visualization method cannot satisfy all of the user requirements or facilitate all types of tasks. Therefore, MetNetGE links the global view of an ontology using ERSF to more traditional visualizations and data representation methods, including indented lists, parallel coordinate plots, and spreadsheets.

### Related Work in Ontology Visualization

This section assesses related work in ontology and hierarchy visualization in terms of the requirements given above. The most widely-used representation for ontology structure is the Windows™ Explorer-like tree view, or indented list (shown in Additional file 1, Figure 2). One implementation of indented list (Class Browser) is evaluated in [16] with three other methods (Zoomable interface, Focus + Context, and Node-link/tree). The indented list lacks the ability to show non-tree edges. Users presented with indented list naturally think the underlining data is a pure tree structure. When asked about non-tree edges in biological data, biologists know that non-tree edges exist, but they do not know how many such edges are in the ontology and only a few know how to find them. This is a typical example where traditional visualization hinders the analysis of data presented to the user.

**Figure 2:**
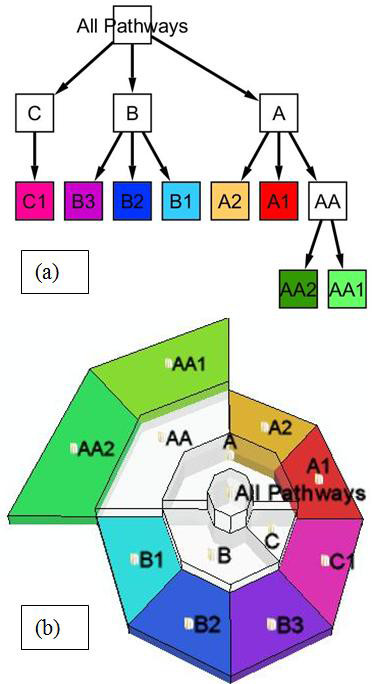
**Visualization of graph G1 with tree structure**. Graph G1 shows hierarchical relationships among leaf nodes (pathways) and non-leaf nodes (pathway categories), drawn in dot layout (left) and the radial space-filling) layout in MetNetGE (right).

Biologists with more computer experience often use specialized Java tools, e.g., MetNetDB's PathTree[17], to map expression data on different ontologies, and then export the file to well established visualization platforms, e.g., Cytoscape [5]. In Cytoscape, they can use well-known 2D layouts, e.g., hierarchic and organic, to view data. We outline the procedure to create visualizations via this method in usage scenario section as a comparison to our approach.

Many methods exist to show the Gene Ontology structure. Thanks to the intrinsic similarity of these two ontologies, these methods can be adapted to show the PO as well. Besides the traditional indented list view used in AmiGO[18], node-link graphs and treemaps [19] are also widely used to visualize GO. OBO-Edit [20] combines an indented tree browser (Tree Editor) and a graphical tree drawing (Graph Editor) which uses the node-link based layout from GraphViz[21]. BiNGO[22], a Cytoscape plug-in for analyzing Gene Ontology, uses the default 2D hierarchic layout from Cytoscape. The node-link based layout is very good at showing simple hierarchical structures (e.g., contain less than 50 nodes). However when the number of entities increases, those layouts become very cluttered and incomprehensible. Additional file 1, Figure 3 shows the result of our PO dataset using these layout methods. We can see that the whole hierarchic structure and non-tree edges are not obvious in these views. Due to the cluttered layout for a large number of entities, researchers normally confine their view to a limited subset of the whole structure, and are thus unable to gain the global knowledge.

**Figure 3:**
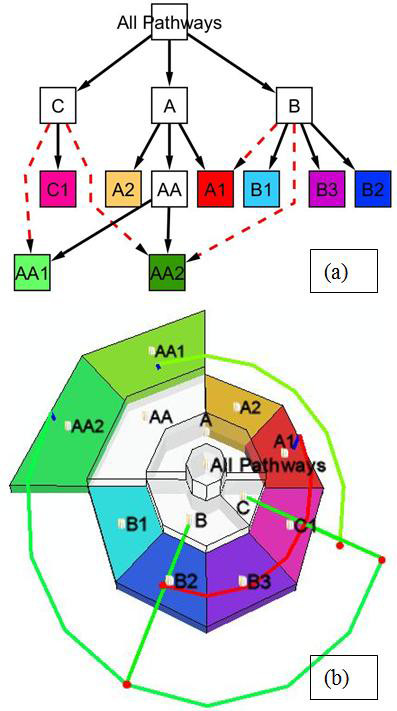
**Visualization of graph G2 with non-tree structure**. Graph G2 drawn using dot layout (left) and the Enhanced Radial Space-Filling (ERSF) layout in MetNetGE using structure-based coloring (right). In the dot layout, green dashed lines represent non-tree edges. In the ERSF layout, yellow orbits with green and blue links represent non-tree relations. For example, the green line extruded from C contains two red-dots: the inner one intersects with orbit of AA1 and the outer one intersects with orbit of AA2. These orbits mean that C is the minor parent of both AA1 and AA2.

Treemap based systems [19] are able to visualize the whole GO with mapped data in one screen, and are suitable for identifying regions of interest. However, the hierarchical structure is hard to see in a treemap since it is a nesting-based layout which overplots the parent nodes with their children nodes [23]. Another limitation of treemap is that it lacks a meaningful representation of non-tree edges, a key requirement. Although Fekete [24] added non-tree edges as an overlay to the treemap, this method created many edge-crossings which made the task of tracing those edges very difficult. As observed in [15], treemaps and other space-filling layouts normally duplicate nodes which have multiple parents. If the node being duplicated is a non-leaf node, the whole substructure rooted at this node will be duplicated as well. Thus duplicating nodes in hierarchic dataset may greatly increase a graph's visual complexity. Duplication also causes confusion for the user. For example, when user finds two regions have similar visual patterns in a treemap, they may think that they have discovered two groups of genes functioning similarly. Unfortunately, they often turn out to be the identical GO terms being drawn twice.

Besides the visualization methods mentioned above, Katifori et al. [15] have also presented many tools and layout algorithms to visualize ontologies and graphs in general. For example, a hyperbolic tree [25] can handle thousands of nodes. However, in a hyperbolic tree visualization, it is difficult to distinguish between tree and non-tree edges among hundreds of edges since they are all represented as links. Another disadvantage is that hyperbolic trees are not space efficient, and normally only a couple of pixels are used for each node. Therefore attributes (like gene expression data) mapped on nodes become hard to distinguish and interpret.

Space-filling methods are considered very space-efficient and are good for mapping attributes on node regions. Despite the disadvantages of rectangular space-filling (such as treemap), evaluations [26] find that radial space-filling (RSF) methods [27] are quite effective at preserving hierarchical relations.

Researchers in economics have utilized 3D RSF to study hierarchical time-dependent data [23]. However, like the traditional RSF and treemaps, their method also suffers the problem in duplicating nodes for non-tree edges. We propose the enhanced RSF (ERSF) algorithm, which uses an intuitive orbit metaphor to explicitly visualize the non-tree edges, and make it appear different than the major hierarchic structure.

### Contributions of MetNetGE

In order to show the overall structure of complete ontologies, MetNetGE provides a space-filling view of the biological network and maps the experimental data onto this view using the Google Earth software and API [28]. MetNetGE is also designed to aid biologists in better understanding complex individual pathways, using a 3D tiered layout, where different entity types and interactions are located on different tiers. MetNetGE allows exploration of new patterns in the data. The contributions of the MetNetGE system to biological data visualization are:

• A 3D tiered layout that shows the main pathway structure and cross layer patterns.

• A novel representation and interaction based on our enhanced radial space filling (ERSF) technique with orbits for visualizing cross-links in an ontology dataset.

• Methods that link the summary statistics of experimental high-throughput data to the ontology visualization.

## Methods

### Implementation

MetNetGE is implemented in Python. The pathway and ontology drawings were created as Keyhole Markup Language (KML) files, and were loaded into Google Earth through its COM API [29]. The graphical user interface (GUI) is written with PyQt [30].

The major research focus of MetNetGE is on summarizing large biological networks on a single display. There are many well-known visualization platforms that are tailored to show biological pathways, e.g., Cytoscape, VisANT, however, none of them can handle the large number of 3D geometries used in the ERSF technique. Google Earth was designed to smoothly handle large 3D geometric datasets. The GE API provides methods for controlling the level of detail and zooming, selection etc. Moreover, GE is a well established and widely used platform with a well-known user interface, thus users may not need intensive training for successful use of MetNetGE.

However, GE also has many limitations when used as an information visualization tool. For example, GE's COM API which we have been using currently does not support dynamic modification and removal of content. As a result, our program can not support many interactive actions like dragging and rearranging the network at this stage.

### 3D Aligned Tiered Layout

MetNetGE features a 3D layout that is both aligned and tiered. Separating the nodes into different layers according to their types (e.g., metabolite, polypeptide, RNA, or DNA) provides a visually-clearer structure on which to superimpose the data.

In the MetNetGE tiered layout, the node placement is based on the results from a user-selected major plane, rather than computing each planar layout independently. The layout of nodes occurs on the major plane first, and then other nodes are set based on their relation to the major plane nodes. For example, for the metabolic pathways, the metabolite layer serves as a natural choice of major plane. In Figure 1, a spring-embedded layout has been selected to first place nodes in the metabolite layer; the algorithm then identifies the nodes in the protein plane that connect to these metabolites so that they line up with the major plane. Next, MetNetGE places nodes that connect to these proteins. Finally, the remaining RNAs and DNAs are placed under the respective polypeptides.

### Visualizing Tree Structure of Ontologies using Radial Space-Filling Methods

To summarize metabolic networks in a meaningful global view, MetNetGE employs the BioCyc pathway ontology, which hierarchically organizes the pathways as a directed acyclic graph, where many parents may point to the same child. For simplicity, we first assume the ontology is a pure tree structure which does not have any non-tree edges, and explain how the traditional radial space-filling (RSF) technique can visualize this simplified biological ontology. The next section shows how the Enhanced RSF algorithm can visualize a biological ontology which contains non-tree edges.

Tree visualization is a widely studied topic. Among all the existing tree visualization techniques, we implement the RSF [27] because it effectively utilizes the screen space and clearly shows the hierarchical relationships between concepts. In addition, in RSF each non-leaf node has its own region, which provides the ability to map cumulative values onto those regions.

To the best of our knowledge, MetNetGE is the first application of 3D RSF in biology and the first algorithm to visualize non-tree edges on a RSF plot.

RSF visualization of a pure tree uses the following rules:

• Each circular region represents one node in the tree. The leaf nodes are placed on the edge of the drawing and the root node is placed at the center. Nodes with the same depth form one layer in the visualization, i.e. the root node forms layer 0, the nodes with depth 1 form layer 1.

• Each circular region has five variables: sweeping angle, depth, radius length, height, and color.

• The sweeping angle of a leaf node is determined by an attribute of the corresponding pathway. For the pathway ontology, we have set each pathway to an equal weight, thus spanning the same angle. For visualizing the gene ontology, it could also be linked to other factors, such as the number of differently expressed genes within a category.

• The sweeping angle of a non-leaf node is the sum of all its children's sweeping angles.

In the initial network view, we use structure-based coloring [27] where the leaf node regions are colored according to the color wheel and the non-leaf node regions are colored as the weighted average of its children's color to convey the hierarchical relationships. The height of each region is set proportional to the height of the subtree rooted at that node. Since color and height only apply to the individual region of each pathway or category, we later use them to map experiment values.

Figure 2a shows a small tree with eight leaf nodes and five non-leaf nodes, labeled as graph G1. Figure 2b shows the result of using RSF in 3D on graph G1. In the PO, Non-leaf nodes correspond to pathway categories, e.g., "A" may represent the category of *acid resistance*, and the leaf nodes represent the pathways that relate to this function, e.g., "A2" may represent the *arginine dependent acid resistance pathway*. In this example, we use a uniform radius length, structure-based coloring, and map the height of the subtree to the region's height.

### Visualize Ontology as Directed Acyclic Graph

As noted earlier, RSF cannot support non-tree edges which are very common in the pathway and gene ontologies. As a result, we developed the Enhanced RSF (ERSF) layout which uses orbits to represent non-tree edges.

This concept is illustrated in Figure 3. Graph G2 (Figure 3a) adds four non-tree edges to G1. The metaphor of "satellite orbits" represents such non-tree edges as circular links. For each child tree node with at least two parents, one orbit circle is drawn on the layer of that node (Figure 3b). The parent that connects the node in the spanning tree is the major parent and other parents are minor parents. The region of each node is placed under the region of its major parent. For every minor parent, a green edge from the center of its region to the orbit of the child is called the 'downlink'. The intersections between links and orbits are called access points which are represented by red dots.

To help users find and trace interesting non-tree edges, the orbits need to be distinguishable from one another. The orbits are first restricted to span in the middle area of each layer, thus leaving a visually apparent gap between orbits in adjacent layers. To distinguish orbits in the same layer, our algorithm puts them at different heights and distances from the center. The orbit with most downlinks is placed as the most distant and highest. This arrangement can help users answer questions like 'Does the *aldehyde degradation pathway *belong to many categories?'.

Coloring strategies also help the user visually distinguish orbits that are located on the same layer. When the orbit color is the same as the child's region, and non-leaf categories' regions are transparent, it becomes easier to see links between regions.

Figure 3 shows that visualizing the ontology using the orbit metaphor have several advantages. First, this design clearly distinguishes between spanning tree relationships and non-tree edges. Second, compared to treemaps with a crosslink overlay [24], there are much fewer edge-crossings. Third, all downlinks of a parent share only one link edge. Thus, the total length of those edges is the same as the length of the longest link. This property reduces the graph complexity, especially when one parent is the minor parent for many other child nodes or a child node belongs to many parent nodes.

### Mapping Experimental Values onto an Ontology

The strategy of a biological scientist evaluating experimental data is to look for which parts of the network show significantly different measurements across different conditions. Questions such as 'Which pathways or categories are most changed under *anaerobic stress*?' can be addressed by mapping the values onto the Pathway or Gene Ontologies.

Since each region of the ERSF contains a group of genes, we use ensemble statistics to represent the overall behavior of the group such as the average or median value of those genes under a certain condition and map it to the color of this region. The height of the region can also be mapped to other values to capture the overall behavior of a gene or set of genes. For example, the height can be set to the average coefficient of variation (CoV) for each gene across a range of experimental conditions:

$$\begin{array}{ccc} CoV\text{​}\;=\;\frac{\sigma }{\mu } & \sigma =\;\sqrt{\frac{\sum_{i=1}^{N}{\left(x_{i}-\mu \right)}^{2}}{N}}; & \mu =\frac{1}{N}\sum_{i=1}^{N}x_{i} \end{array}$$

MetNetGE uses animation to show the values of a series of experiments. For instance, a time-series experiment with 7 time points is presented as an animation of 7 frames. Users can use either the time controller from Google Earth or the animation control panel in MetNetGE to control the animation.

Differentially expressed can also be mapped directly on the ontology drawing. The user first defines a threshold based on the experimental data, e.g., 0.7 fold-changes is the default suggested by collaborating biologists. Every gene with a value change more than the threshold is considered differentially expressed. A differentially expressed gene is down-regulated if the treatment value is lower; otherwise it is up-regulated.

Another important value for biologists is the statistical significance of the observed differences in the experimental data. Simple analyses of experimental data, such as p-values for over-representation of PO terms, can be calculated using Fisher's exact test. One typical working scenario is: identify a list of genes of interest that are differentially expressed between conditions, or are of particular interest; calculate p-values of over-representation of pathways and categories using a Fisher's exact test; visualize these p-values on the ontology drawing.

## Results

This section demonstrates the use of MetNetGE through illustrations of how the visualizations work along with the results of a pilot user study for the ERSF visualization of the pathway ontology. A use case comparing the standard methods for analyzing microarray expression data using ontology show the effectiveness of the method for providing a global understanding.

### 3D Tiered Layout

In this section, we present the 3D Tiered layout for *Arabidopsis *pathways. Two examples illustrate how MetNetGE enhances insight in both signaling and metabolic pathways. Figure 1 shows the *ethylene biosynthesis and methionine cycle *in *Arabidopsis *in both Cytoscape and MetNetGE. The 3D tiered layout in MetNetGE shows a clear circular structure in the top metabolite layer which illustrates the flow of mass through the metabolic cycle. The three blue edges from the protein layer indicate that three protein complexes catalyze the metabolic reactions. This structure separates out the signaling control from the metabolic flow very effectively. In Cytoscape, the same pathway is more difficult to interpret as it combines metabolic flow and regulation in a complex network.

The same pathway structure is also shown in Additional file 1, Figure 4 but arranges nodes in each layer independently. We can see that there are more edge-crossing when the drawing is projected to the 2D viewing plane, and the pattern of transcription and translation we observed in Figure 1 is not obvious anymore. As a result, when compared with existing layered layout who arranges nodes in each layer independently, our novel layout approach produces graphs that show a clearer structure.

**Figure 4:**
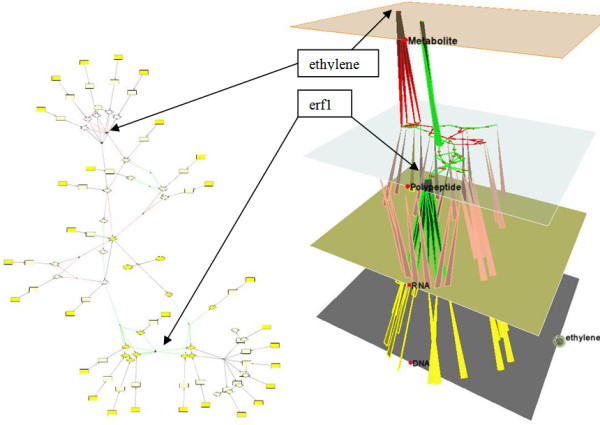
**Visualize one signaling pathway**. The pathway 'ethylene signaling' is viewed using the yFiles Organic layout in Cytoscape (left) and the 3D tiered layout in MetNetGE (right). The 3D tiered layout reveals several interesting features which can not be easily seen from the 'Organic' layout. For example, there are two metabolites (ethylene and ATP) that regulate many proteins, and one protein (erf1) that activates many RNAs.

Figure 4 shows the *Arabidopsis ethylene signaling*. The MetNetGE 3D tiered layout shows how one metabolite (*ethylene*) and one protein (*erf1*) have many regulation links to other layers. This linkage is not obvious in the Cytoscape view.

### Use Case Comparison of ERSF and Standard Methods

This section compares how biologists can work with *E. coli *Pathway Ontology using an experiment on BaeSR [31] from the *E. coli *gene expression database, M3D [32]. This case study compares a traditional workflow to the usage of the ERSF in MetNetGE to highlight differences between the approaches.

### Exploring Data with Traditional Methods

The standard working method for each of our test biologists is to use three tools together: Microsoft™ Excel, PathTree [17], and Cytoscape [5]. The PO is recorded in PathTree, and is represented as an expandable indented list similar to that shown in Additional file 1, Figure 2. The gene expression data is recorded in Excel files, one row for a gene, and one column for a condition. The summarized data for PO is also stored in an Excel file, where each row represents one pathway/category and each column shows one value, e.g. average gene expression or p-value.

To view the ontology structure, users need to export the PO to an XML file that is loaded into Cytoscape. Users can then use Cytoscape's layout to arrange the nodes automatically (normally using hierarchic or organic layout). However, due to the limitations of these node-link based layouts, the visual result is not comprehensible for the whole ontology (see Additional file 1, Figure 3). In addition, the indented list is not linked with Cytoscape which makes searching category or pathway difficult. However, this difficulty can be overcome by writing PathTree as a plugin for Cytoscape in the future.

Although the node-link based layout can show the non-tree edges in PO, those edges are buried in the cluttered edges, and are difficult to detect. In order to trace the related ontology terms of a given node, users need to make the surrounding edges non-overlapping by manually moving the nodes around in the local region. Due to the difficulty of those operations, users normally export only a small subset of the whole PO, e.g. only pathways from a specific category such as biosynthesis. The normal size of the output network is between 10 to 50 nodes.

As illustrated in Additional file 1, Figure 5, users can load expression data into PathTree, and then set a criterion to filter out unwanted ontology terms. For example, users may set the filter as "p-value < 0.01". Then only those statistically significant nodes will be loaded into Cytoscape (around 60 in this dataset). These nodes are often disconnected from each other. Viewing the interesting pathways alone is important. However, it loses the surrounding biological context. Thus, important questions like "tell me categories that have at least *N *pathways and at least half of them are statistically significant" cannot be answered.

**Figure 5:**
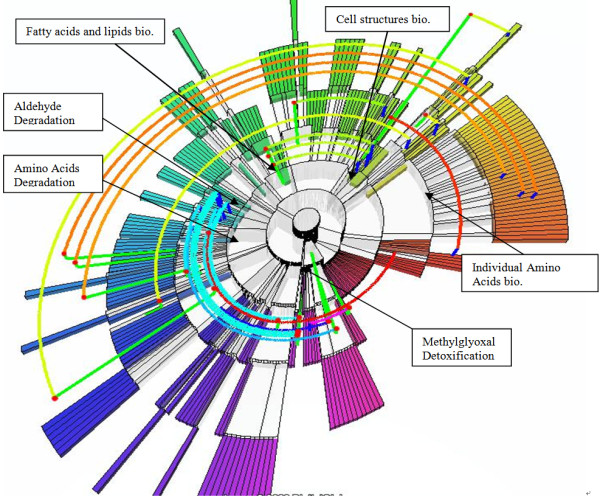
**Pathway ontology shown with proposed ERSF layout**. The hierarchical structure of the ontology is clear in this view. The depth of the ontology tree, six, can be seen by counting the number of rings in the display. There are many pathways that belong to at least two categories, e.g., three pathways from *individual amino acids biosynthesis *(on right) also belong to the category *amino acids degradation *(on left). Also many pathways from *aldehyde degradation *(on the left of 3rd layer) belong to the category *methylglyoxal detoxification*. This kind of multiple inheritance information is difficult to see in other visualization methods.

### Exploring Data with ERSF using MetNetGE

Using the same ontology data from *E. coli*, MetNetGE presents the user with the ERSF view shown in Figure 5. It is clear that the most orbits are concentrated on the third layer, and one category (*methylglyoxal detoxification*) contains many children in other categories because its green uplink intersects many light blue orbits.

**Figure 6:**
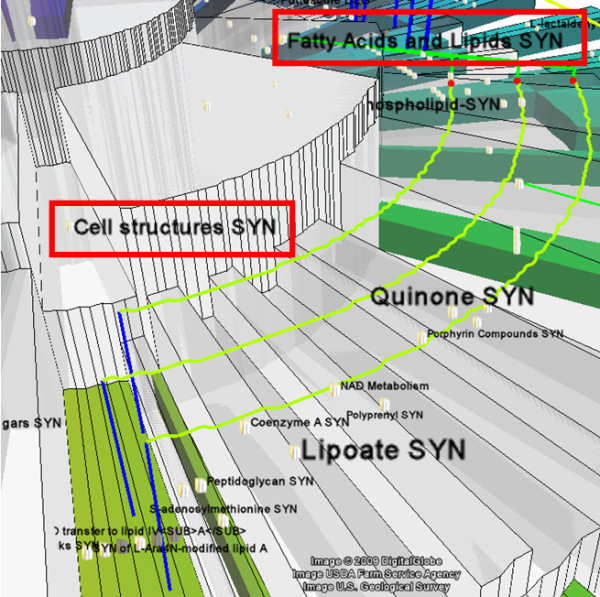
**Zoom in view of pathway ontology**. When zoom in, the labels of categories are shown, e.g. three pathways under *cell structures biosynthesis *also belongs to *fatty acids and lipids biosynthesis*.

It is also clear that three pathways in the category *cell structures biosynthesis *are also the children of another category *fatty acids and lipids biosynthesis*. When a user wants more information about those non-tree edges, he can rotate and zoom the view, and get results similar to Figure 6.

**Figure 7:**
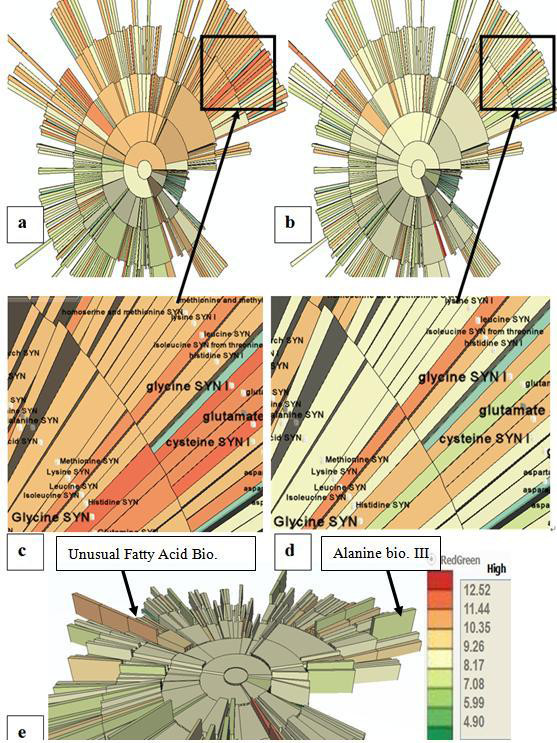
**Experimental values mapped onto the pathway ontology**. Average expression values are shown for each condition. The color of each region reflects the differential expression for these pathways. The orange and red colors in condition 1 show that these categories have much higher expression values in condition 1 (a) than in condition 2 (b). The differences between these two conditions are more obvious when using animation in MetNetGE. (c)(d) show the details in the *glycine biosynthesis *categories. When the view is tilted (e), the categories with high Coefficient of Variation (CoV) is shown by their higher height.

To analyze the gene expression data, we initially map the average expression value on color, and map the CoV on height. The visual results of two frames are shown in Figure 7a and 7b. The first frame shows the value of one replicate in controlled condition, while the second frame shows that of the treatment condition.

Users can tilt the view to see the height of each region (Figure 7e). In this view, one category (*unusual fatty acid biosynthesis*) stands out, because it and its descendants have very high CoV and expression values. This discovery demonstrates the benefit of using 3D to show these two attributes together. Another similar interesting discovery is the pathway *alanine biosynthesis III*, which also has very high CoV but very low expression values.

By switching between these two conditions, we notice that most of the pathways and categories have a greenish color under the treatment, which indicates lower expression values in the treatment condition than in the controlled condition. This is an interesting trend, since in most experiments the treatments normally have greater values. To confirm this trend, we can map the difference between these two conditions directly on the ontology.

**Figure 8:**
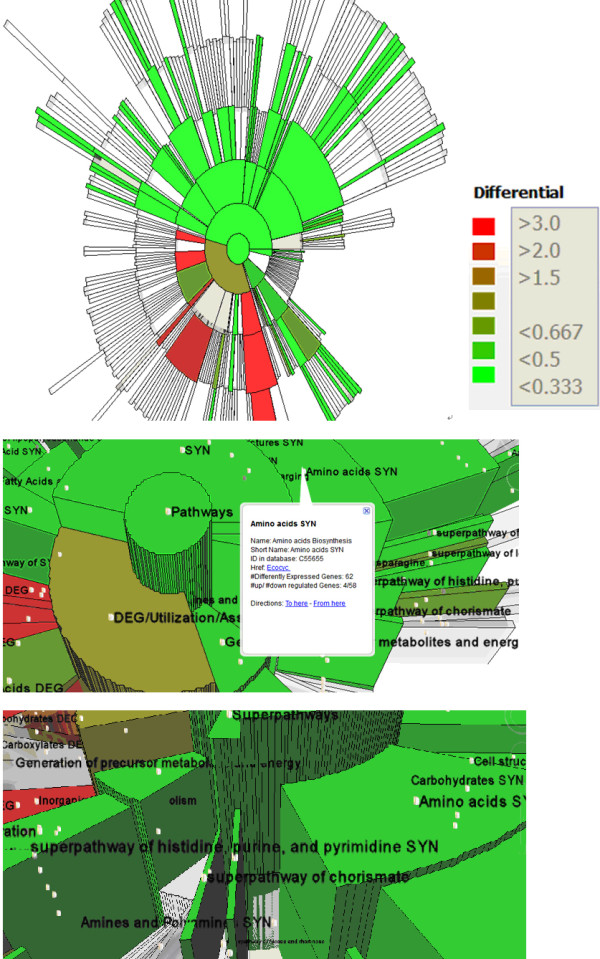
**Differentially expressed genes mapped on the pathway ontology**. Color indicates the ratio of up/down regulated genes; height shows the log value of total number of differentially expressed genes. Most of the upper side of ontology categories is down-regulated, while some categories on the lower-left side are up-regulated. Zoomed in view (center) shows the detail of amino acids biosynthesis. (bottom) shows that two superpathways (histidine, purine, and pyrimidine biosynthesis, and chorismate) have many more genes differentially expressed downward than other superpathways.

Additional file 1, Figure 6 shows the results from the original analysis on this data set [31], where each black/grey bar shows one category. This representation clearly shows the properties of the listed dozens of categories, however it misses many other categories and loses the hierarchical relations among them. MetNetGE provides a "differential view" (as in Figure 8) to facilitate this task by mapping the logarithm-base 2 value of the number of differentially expressed genes onto region's height. he logarithm helps accomodate the large dynamic range for this value (from very low to very high cumulative value of the top categories).

To reflect the up/down regulation property of an entire category or pathway, the ratio of the number of up-regulated genes to that of down-regulated genes is calculated and mapped onto the region's color. As a result, the reddish regions in are mainly up-regulated while greenish regions are mainly down-regulated. Other non-interesting regions (ones with few genes differentially expressed) are left transparent to let the interesting ones stand out. It is clear that there are more interesting regions than the dozens listed in supplemental Figure 6, and their relationships can be seen. We also confirmed the hypothesis that most categories are mainly down-regulated since most of the regions are greenish.

After tilting the view (as in the bottom of Figure 8), shows that among 12 superpathways, only two have many differentially expressed genes. Clicking one of these, *superpathway of chorismate*, gives a pop-up dialog with information about this pathway, and shows its details in the linked indented list. To get more information about the genes in this pathway one can select the "add genes" button, to add all its genes to a spreadsheet table and a parallel coordinate plot.

To enable biologists to create various views of data, MetNetGE provides customization of the mappings of color and height of each region. Moreover, biologists can easily switch between different views to gain combined knowledge.

In summary, the "differential view" approach (Figure 8) can help biologists answer critical questions like: which pathways or categories have many genes differentially expressed, or is one particular pathway mainly up-regulated?

### Pilot User Testing

We conducted a qualitative pilot user test involving four users. The goal was to better understand the needs of the biologist-users and to test the effectiveness of the ERSF and MetNetGE. Users were presented with several tasks in two categories following the analysis procedure presented in the ERSF use case presented above. The goal was to see how well the users understood the ontology structure and what pathways were affected by the gene expression data. The users worked in a relaxed setting where the tasks were not timed. Users worked through those tasks with assistance as required on both the traditional method and MetNetGE, and were encouraged to think aloud during the whole procedure. The users were interviewed at completion to determine preferences and their overall impression. Among the four users, two are postdoctoral biologists in plant research, one is a graduate student in the program of Bioinformatics and Computational Biology, and one is a graduate student in Computer Science who has been involved in a biology-related project.

All users who participated in the pilot user test preferred the MetNetGE solution to the traditional one. The users cited the ability to show the whole ontology structure and see the relationship between concepts as an important feature. Visualizing the entire network is expecially useful when viewing a system-scale experimental dataset. Users found this difficult when they are viewing a small subset of the system at a time. Moreover, users generally gave up on some time-consuming tasks. For example, finding the pathways that belong to at least two categories is extremely difficult using indented lists and node-link based layouts.

The user testing also exposed a critical drawback of structure-based coloring. Users thought that the red color regions around zero degree should represent similar types of pathways, but they turned out to be pathways in completely different categories. Similarly, the spatial proximity of adjacent pathway categories led users to believe that they are similar, but in many cases they are not. As a result, a future improvement of the layout will be to give sufficient blank space between each category and use distinct color mappings for some major categories.

Animation proved useful to show trends in time-variant experiments. However, users said that the ability to rearrange a sequence of conditions and quickly switch back-and-forth between two conditions is much more important. MetNetGE provides this ability with a simple table containing all the conditions, thus users can easily click the name of the condition to see its data mapping.

When users worked with MetNetGE, the team noted that although ERSF provides a 3D view of the ontology, users mostly viewed it from the top down orientation, which is essentially a 2D ERSF layout. Therefore, when users were given the choice to map an attribute to either color or height, all of them prefered mapping the most important attribute to color. Some possible reasons include: biologists are used to traditional 2D tools, and height is hard to interpret precisely due to foreshortening [33]. Nevertheless, the 3D view provides the benefit of mapping two variables simultaneously (color and height). This ability is important for some tasks that may lead to interesting discoveries, e.g. finding pathways that have high CoV and high expression value.

Users in our pilot study were eager to convert their own statistical analyses onto the ontology and proposed many new statistical approaches that would be useful. For example, besides the Fisher exact test currently provided by MetNetGE, users requested other models to calculate statistical significance of ontology terms. To facilitate this request, MetNetGE allows importation of a list of genes or statistical test results in a simple comma-separated-values (CSV) format. Users can then use their own data to generate values via statistical tools in R or exploRase [34], and visualize these on ERSF drawings.

Another drawback of MetNetGE is the lack of interactive modification of the drawing. Since Google Earth's COM API currently does not support dynamic creation and deletion of KML content, MetNetGE is constrained to show only static drawings and framed animations.

### Case Study with *Arabidopsis *Pathway Ontology

**Figure 9:**
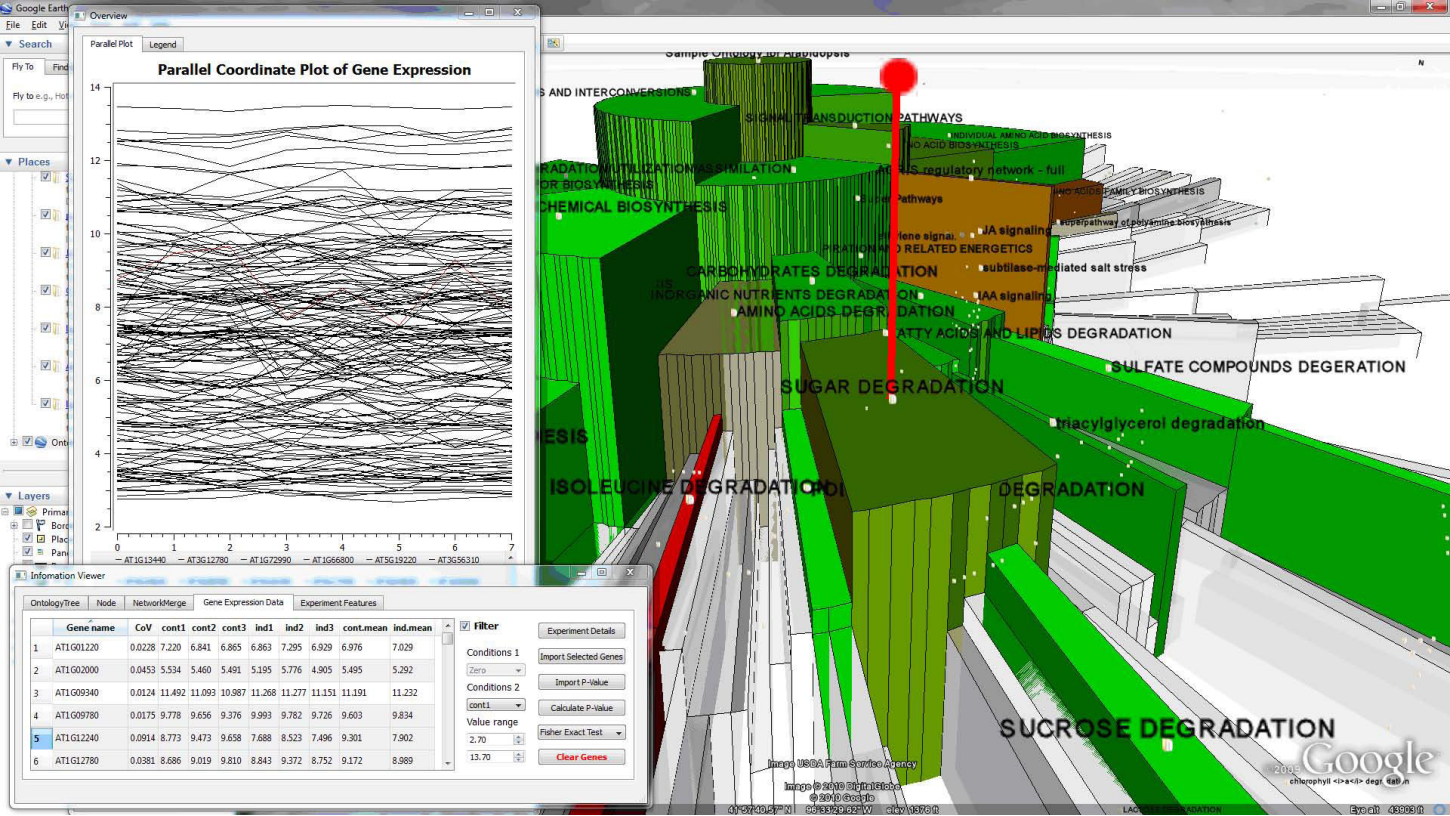
**Arabidopsis Pathway Ontology with experimental data**. The pathway ontology containing more than 4 differentially expressed genes (difference >0.5) are shown in green (lower in transgenic line) or red (higher in transgenic line). The pathway ontology of *sugar degradation *was found down-regulated in transgenic line which was not found in previous analysis. By selecting the category of interest (*sugar degradation*), it is able to see the expression data in that category with parallel coordinate plot.

One example study in our group is to identify differentially expressed transcripts in transgenic Arabidopsis that were genetically engineered to accumulate PHB (polyhydroxybutyrate) compared to control plants [35]. In this study, author found 103 transcripts are differentially accumulated at q-value cut off <0.05 [36] With the list of 103 genes, only two GO terms, light-harvesting complex (cellular component, GO: 0030076) and photosynthesis (biological process, GO: 0015979) are found to significantly changed using Fisher's exact test (p-value < 0.001) [35]. The data is then loaded to MetNetGE without filtering out using statistical test. Six datasets from 3 replicates of 2 genotypes are loaded into MetNetGE. Among 22810 genes on the microarray, 1966 genes were mapped to Arabidopsis pathway ontology database from MetNetDB (http://www.metnetDB.org). Figure 9 shows the pathway ontology view with data using mean expression values of each genotype. In this view, pathway ontology containing differentially expressed genes are shown clearly and can be freely explored. Animation of continuous view of each replicates and genotypes allows user to see and explore the data without loosing information in averaging replicates.

The MetNetGE analysis found that some pathway ontology terms including *sugar degradation *are down-regulated in transgenic line that was not found in previous analysis (Figure 9). By selecting that ontology term, we were able to see the data from 163 genes in this ontology with parallel coordinate plot. Some genes which were clearly down-regulated (e.g. AT1G12240) were not found in previous analysis as their p-values were not small enough to be in the selected list.

## Conclusions and Future Work

This work is motivated to find methods that help biologists understand changes in system-wide datasets in large metabolic networks. The key step is using existing controlled vocabularies such as the Plant Ontology and the Gene Ontology to structure the data in a metabolic pathway or a functional category context. The proposed ERSF algorithm provides easy visual identification and navigation of non-tree edges in an ontology and it allows large scale experimental data to be mapped and navigated on the context of the hierarchical structure of the ontology, which may lead to discoveries on a system level. To facilitate the study of system-level experimental data, multiple types of summary statistics can be mapped onto the ERSF visualization to characterize the behavior of groups of genes. The ERSF method is best suited for visualizing medium-sized multivariate hierarchic data (contains 100 to 1000 nodes) and with multiple inheritances.

MetNetGE also uses the 3D Aligned Tiered Layout to visualize individual pathways. This layout groups nodes into distinct layers based on node type or sub-cellular location. Instead of generating layouts for each layer independently, the nodes' positions are established on one major plane, and then the positions of other nodes are computed. This layout helps the user visualize cross-layer patterns as well as helping the main metabolic reactions to stand out. One of our ongoing works is to improve this layout to visualize more complex pathways which may contain both metabolic and signaling reactions. The natural distinction of layers can help distinguish the mass flow from the signaling and regulation in complex pathways.

Pilot user testing shows that users prefer using the global ERSF approach to their current working solutions, which have been based on indented lists and node-link layouts. Future works will include evaluations of representations for non-tree edges along with linked views of the data including pathways and statistical plots. Initial user testing shows that using an ontology structure linked to statistical graphics is powerful in real world usage. Therefore, a larger quantitative user study about the effectiveness of ERSF and the linked views is planned in the near future.

Since Google Earth's COM API currently does not support dynamic creation and deletion of KML content, MetNetGE can show only static drawings and framed animations. MetNetGE will be ported to JavaScript to utilize the Google Earth API [37], which not only permits dynamic content creation, but also supports other operating systems and can be viewed from browser.

## Availability and requirements

Project name: MetNetGE

Project home page: http://www.metnetge.org

Operating systems: Windows Only. Since Google Earth COM API only supports Windows systems, our program can not be used on Mac OS, Linux and any other operating systems.

Programming language: Python

Other requirements: Python 2.5 or higher; Google Earth, PyQt and other required libraries (listed in the documentation on project home page)

License: Freely available under GNU GPL license.

Restrictions to use by non-academics: None

Source code: The program's source codes are submitted as the additional file 2.

Demonstration videos: The video for 3D tiered layout is submitted as the additional file 3. The video for viewing pathway ontology is submitted as the additional file 4.

## Authors' contributions

MJ was the main developer of the tool. He also proposed the 3D aligned tiered layout and ERSF layout. SC proposed to map the differentially expressed genes with p-values on the ontology drawing, and tested MetNetGE with biological data. DR designed the framework of using PyQt and other libraries to build the program. ESW specified the requirements of the tool for biologists. JAD was the lead of the project and had the initial idea of using Google Earth to visualize biological data. All the authors read and approved the final manuscript.

## Supplementary Material

Additional file 1 **Supplemental pictures**. This file contains 6 supplement pictures that help to illustrate the strength and unique features of MetNetGE. Click here for file

Additional file 2 **MetNetGE source code**. This is the source code of MetNetGE. Please go to MetNetGE.org to find the latest version, dependency packages and tutorials. Click here for file

Additional file 3 **Demonstration video 1**. Part 1: 3D tiered layout. Click here for file

Additional file 4 **Demonstration video 2**. Part 2: View Pathway Ontology and Experimental Data. Click here for file
