# Supplementary material for: MetNetGE: interactive views of biological networks and ontologies
Source: BMC Bioinformatics. 2010 Sep 17;11:469. doi: 10.1186/1471-2105-11-469 (PMC2946353; doi:10.1186/1471-2105-11-469)

Supplemental Figures

Figure 1 Metabolic network of *Arabidopsis* from MetNetDB.

Metabolic network with 15,066 nodes and 17,993 edges, as viewed in Cytoscape with the 'Organic' layout. Many pathways are highly interconnected to form a network visualization which is impossible to interpret.

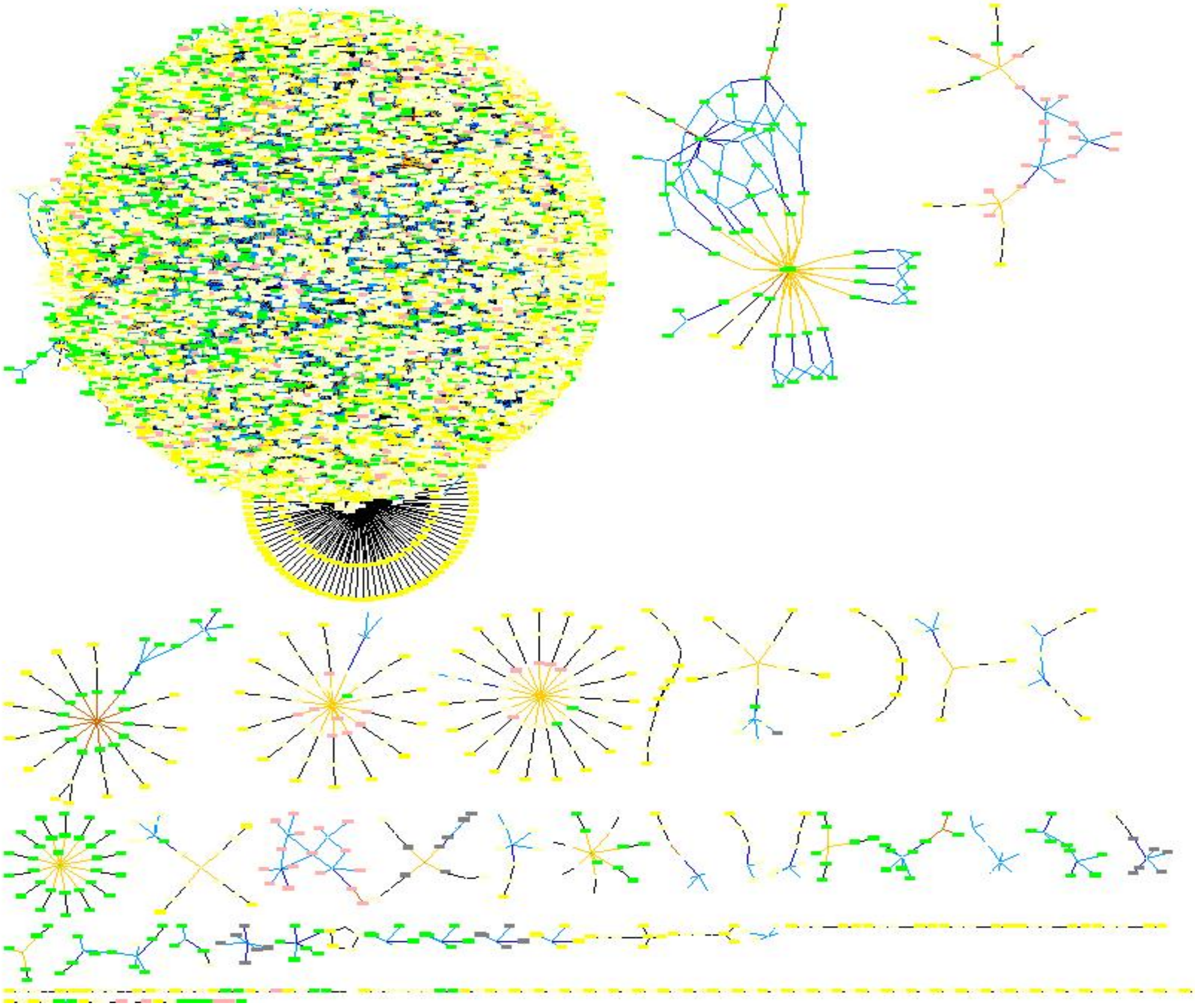

Figure 1 ***E. coli*** **Pathway Ontology.**

The E.coli Pathway Ontology from the EcoCyc website is shown with the traditional indented list view. All the categories are collapsed except Detoxification.

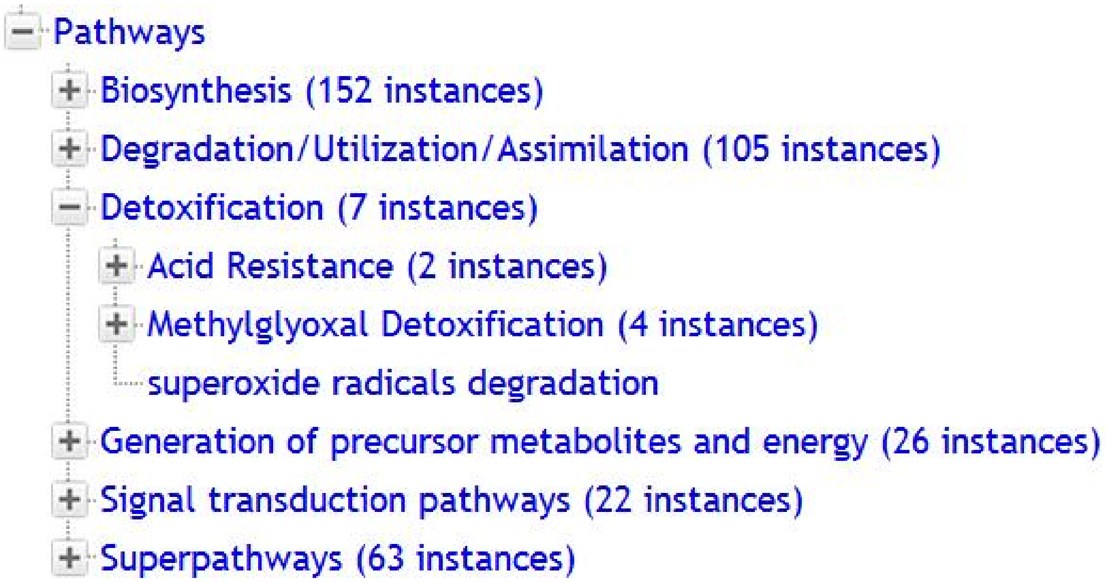

Figure 3 Pathway ontology shown in Cytoscape.

The pathway ontology of *Ecoli* from EcoCyc is shown in Cytoscape using circular layout (top left), organic layout (top right), and hierarchic layout (bottom). The ontology contains 442 nodes and 508 edges. The hierarchical structure can hardly be seen.

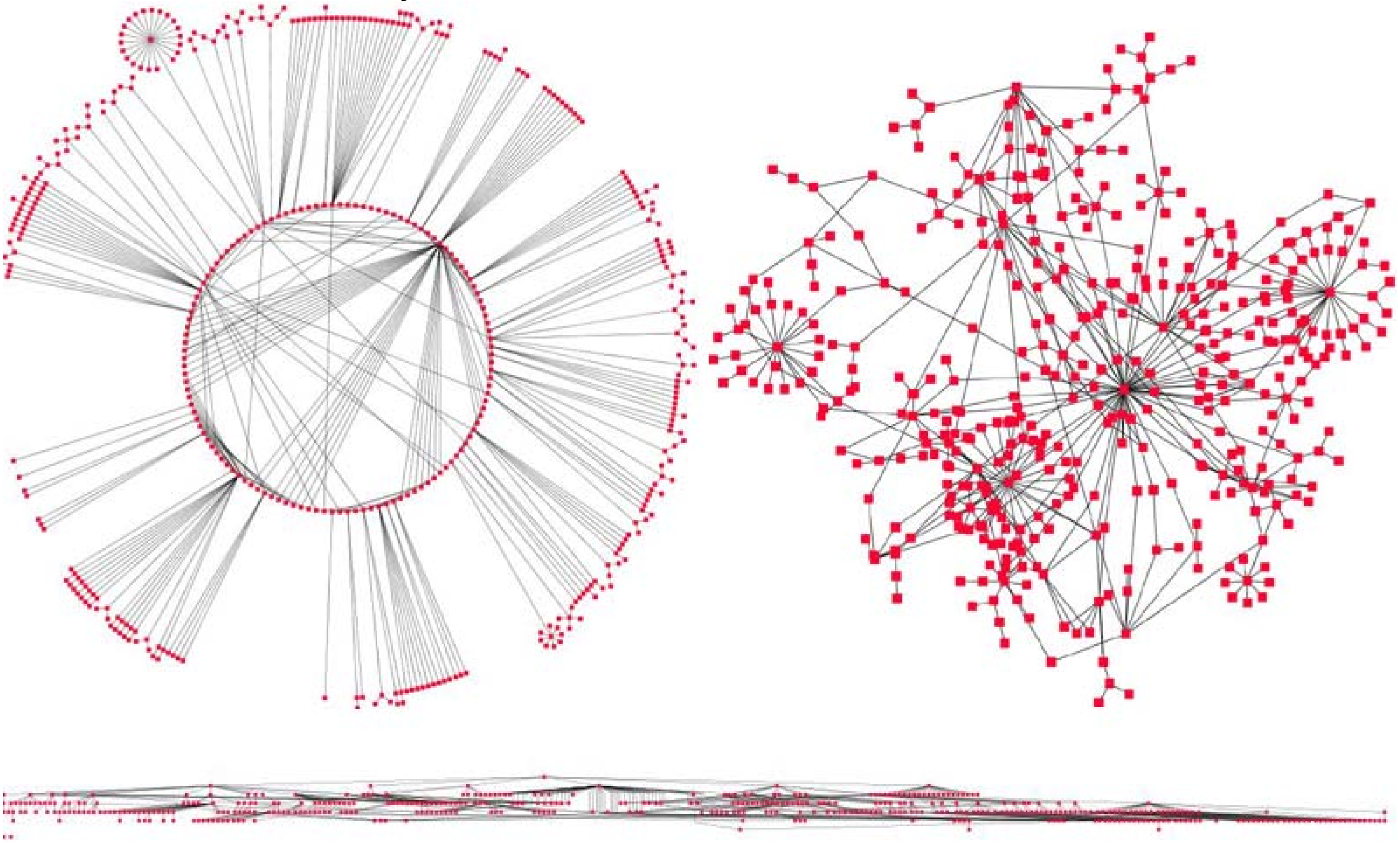

Figure 4 Layer independent layout.

The pathway *ethylene biosynthesis and methionine cycle* is drawn using 3D layered layout. However, similar to Arena3D, we calculate each layer's layout independently. The resulting graph is harder to interpret and the pattern of transcription/translation is not obvious anymore.

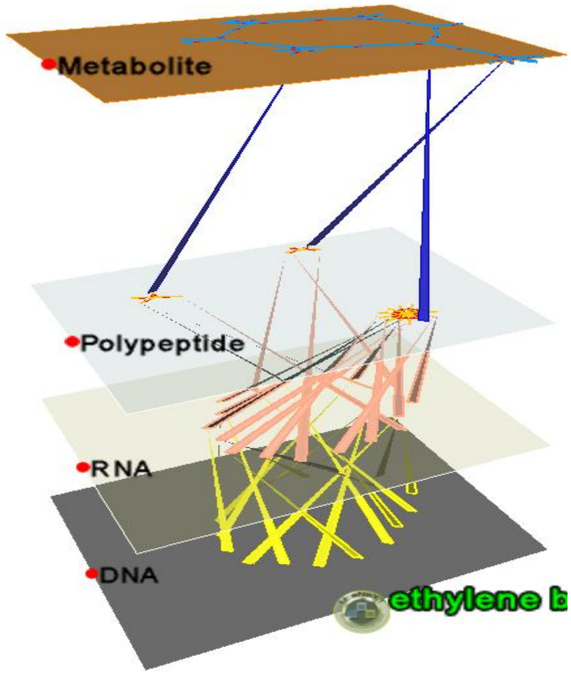

Figure 5 The traditional method used by our biologist users..

PathTree (left) uses indented list and Cytoscape (background) uses hierarchic layout. Due to the limitation of these two methods, only a small subset (around 60 nodes) of the whole ontology is shown.

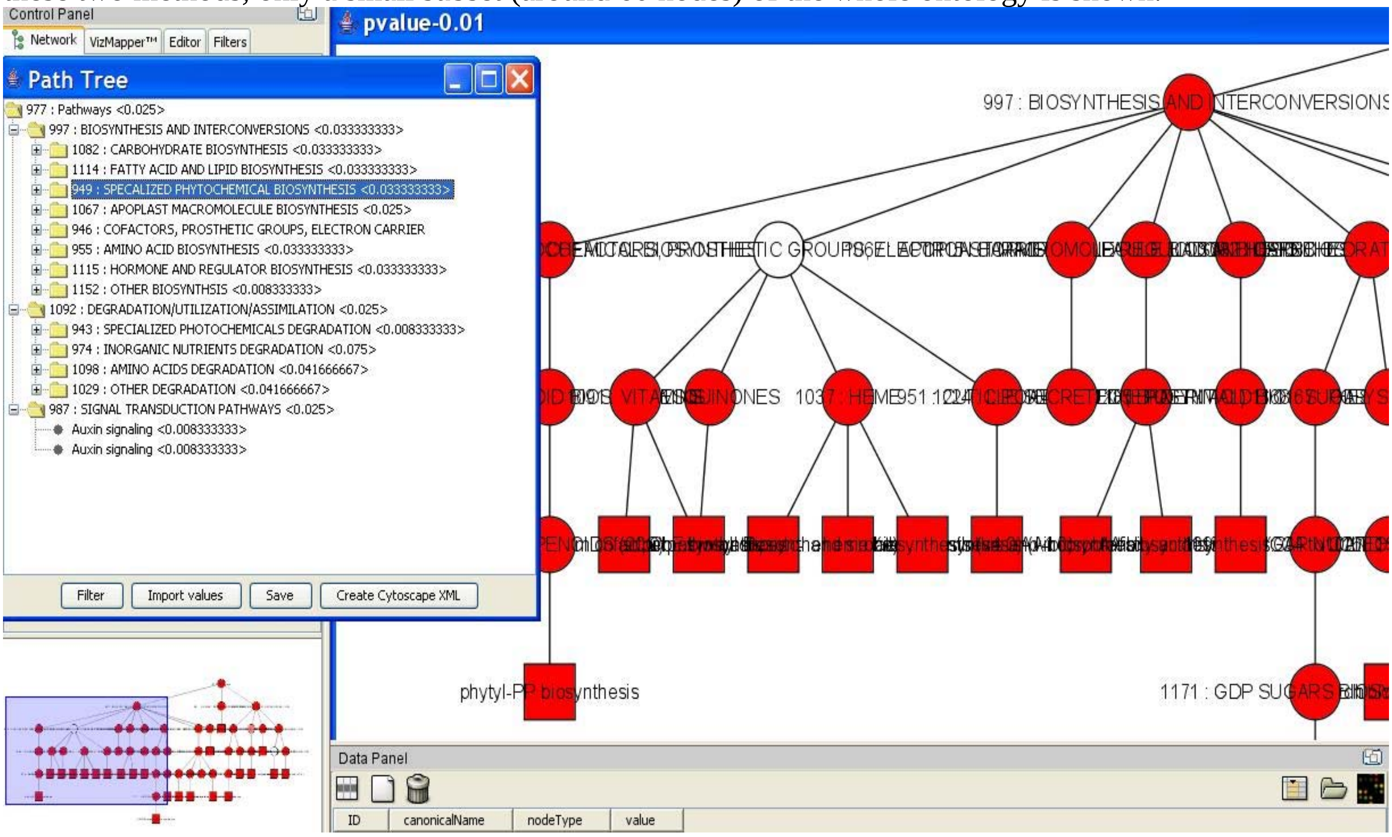

Figure 6 Original results of M3D data.

The length of the bar represents the total number of genes that are differentially expressed while up and down regulated genes are shown in black and grey, respectively. Two treatments are compared. Although this representation clearly shows the properties of the listed dozens of categories, it misses many other categories and totally loses the hierarchical relations among them.

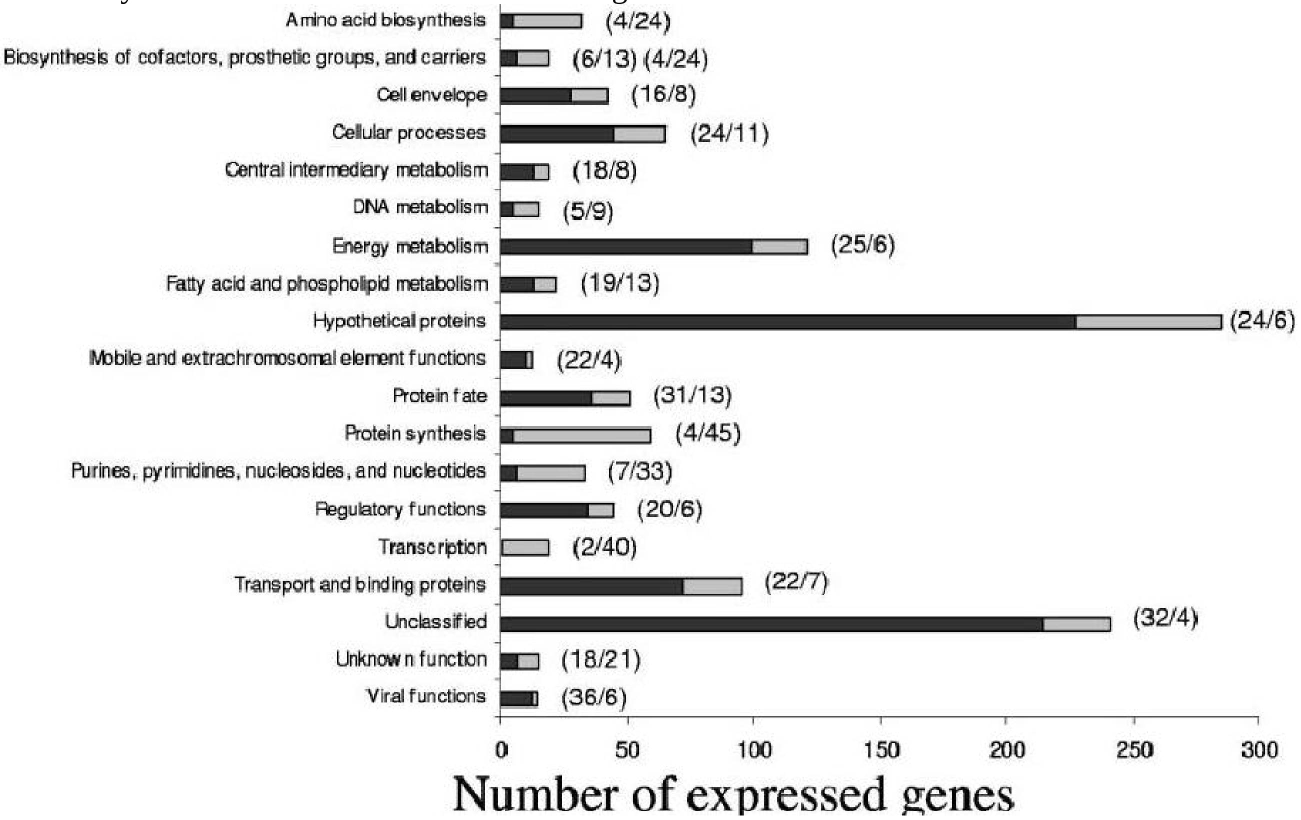

Supplement: Additional file 1 — Supplemental pictures. This file contains 6 supplement pictures that help to illustrate the strength and unique features of MetNetGE. [file 1471-2105-11-469-S1.PDF]
