# Supplementary material for: MetNetGE: interactive views of biological networks and ontologies
Source: BMC Bioinformatics. 2010 Sep 17;11:469. doi: 10.1186/1471-2105-11-469 (PMC2946353; doi:10.1186/1471-2105-11-469)
Supplement: Additional file 2 — MetNetGE source code. This is the source code of MetNetGE. Please go to MetNetGE.org to find the latest version, dependency packages and tutorials. [file 1471-2105-11-469-S2.ZIP › MetNetGE-1.0.3/Readme.htm]

Feature List (For Ontology View)


**Start
the program:**

Double click mge\_start.pyw,
or mge\_start.py

 

**Feature
List (For Ontology View)**

1.      
Show Test Ontology: press ¡®View
Ontology¡¯, the ontology should be shown, and you are fly
to the top of a color pie.

2.      
Add Genes to the ontology:
press ¡®Add Genes¡¯ in main window. You can confirm by seeing #Genes column in
ontology table

3.      
Show expression data. Choose
the data combobox, default is M3D-7, then press ¡®Data File¡¯. After a while,
the pie changes to a tilted and colored version. Also, the ontology table has a
column named ¡®#Genes w/ Epxr¡¯, etc. If you choose ¡®CustomData¡¯, load a data file in a format in Appendix A.

4.      
View detailed gene expression
values. Click the pathway or class contains the genes you are interested in the
table, press ¡®Add Genes¡¯. Then overview window will open, and you can also see
gene info in ¡®Node¡¯ tab and ¡®Gene Expression Data¡¯ tab.

5.      
Save the gene node list. Click
¡®File¡¯->¡¯Export¡¯->¡¯Gene Node List¡¯, or click ¡®Export Node List¡¯ in the
¡®Node¡¯ tab, choose a file name, then save.

6.      
View animation. Click play
animation tool in GE (upper left)

7.      
Review the ontology. Click
¡®Reset¡¯.

8.      
Show ontology related to a
selected region. Press ¡®Show relate¡¯, and use ¡®Show all¡¯ to recover.

9.      
Brushing. When click in table
or tree, the corresponding region in GE will be automatically pointed by a red
arrow. When double clicked, you will fly to the region. When click a icon in GE, press ¡®Show in Table¡¯, will highlight the
parent in tree, and itself in table.

 

**Feature
List (For Pathway View)**

1.      
Show the default pathway. In
the mainwindow, select pathway view tab, click ¡®Auto
Load¡¯. Wait for GE to open, and the pathway is shown by default 3D layer
layout.

2.      
Choose a pathway to view. Select
Data Source first, then click ¡®Open XML¡¯ button, and select the file you want.
Then after the small progress bar go to 100%, click ¡®View Pathways¡¯

3.      
Change layout. After loading
one pathway, choose the layout radio to the intended category, then choose the
layout in the combobox, and then press ¡®Apply¡¯
button.

 

**Appendix
A: Custom Gene Expression data format.**

Tab deliminated.

l  First line is header:

l  First column is probe\_id.

l  Second column is gene name.

l  From 3 third column to the end, is
expression data.

b#     name 4uM DeaNO       8uM DeaNO       SeOH

b0001         thrL   0.0766366  0.0122437  0.0240628

b0002         thrA  0.00808551         -0.00787339        -0.0429918

b0003         thrB  0.0720004  -0.00401984        -0.0478384
