# Supplementary material for: MetNetGE: interactive views of biological networks and ontologies
Source: BMC Bioinformatics. 2010 Sep 17;11:469. doi: 10.1186/1471-2105-11-469 (PMC2946353; doi:10.1186/1471-2105-11-469)
Supplement: Additional file 2 — MetNetGE source code. This is the source code of MetNetGE. Please go to MetNetGE.org to find the latest version, dependency packages and tutorials. [file 1471-2105-11-469-S2.ZIP › MetNetGE-1.0.3/SampleData/SuhYeon/Tannins.pdf]

Applied and Environmental Microbiology, Jan. 2008, p. 535–539 Vol. 74, No. 2
0099-2240/08/$08.00+0 doi:10.1128/AEM.02271-07
Copyright © 2008, American Society for Microbiology. All Rights Reserved.

The BaeSR Two-Component Regulatory System Mediates Resistance to Condensed Tannins in *Escherichia coli*▽†

Erwin G. Zoetendal,[1]‡ Alexandra H. Smith,[2]* Monica A. Sundset,[1,3] and Roderick I. Mackie[1]

Department of Animal Sciences and Division of Nutritional Sciences, Institute for Genomic Biology, University of Illinois at Urbana-Champaign, Urbana, Illinois[1]; Agtech Products Inc., Waukesha, Wisconsin[2]; and Department of Arctic Biology and Institute of Medical Biology, University of Tromsø, 9037 Tromsø, Norway[3]

Received 5 October 2007/Accepted 13 November 2007

The gene expression profiles of *Escherichia coli* strains grown anaerobically with or without *Acacia mearnsii* (black wattle) extract were compared to identify tannin resistance strategies. The cell envelope stress protein gene *spy* and the multidrug transporter-encoding operon *mdtABCD*, both under the control of the BaeSR two-component regulatory system, were significantly up-regulated in the presence of tannins. BaeSR mutants were more tannin sensitive than their wild-type counterparts.

Condensed tannins, or proanthocyanidins, are common secondary metabolites of plants, consisting of flavonol polymers (17). High concentrations of tannins in fodder plants inhibit gastrointestinal bacteria (6, 9, 10, 20) and reduce animal performance (9, 18, 21, 23). Numerous mechanisms of how tannins may inhibit bacteria have been proposed, including tannin-polymer complexation, tannin-induced membrane disruption, and metal ion chelation (22). Despite the antimicrobial activities of tannins, many tannin-resistant bacteria have been isolated. However, the mechanisms behind this resistance are still unknown (22). Gram-negative bacteria may be less sensitive to the inhibitory effects of tannins, as gastrointestinal bacterial populations shifted toward gram-negative *Enterobacteriaceae* and *Bacteroides* species in rats fed diets containing 0.7 to 2.0% condensed tannins (20). There was a corresponding decrease in the gram-positive *Clostridium leptum* group. *Escherichia coli* is resistant to concentrations of up to 1% *Acacia mearnsii* (black wattle) tannin extract (WTE) under anoxic conditions. However, it was demonstrated to be sensitive to 0.1% under oxic conditions due to auto-oxidation of tannins resulting in hydrogen peroxide generation (19). To get insight into the mechanisms responsible for resistance to condensed tannins under anoxic conditions, we conducted a gene expression study to measure the total transcriptional responses of *E. coli* in the presence of WTE.

The *E. coli* strains used in this study are described in Table 1. All growth experiments were performed with MOPS (3-morpholinopropane-1-sulfonic acid) medium (12), with glucose (0.4%) as the carbon source, and iron and trace elements were provided by trace element solution SL-10 (Deutsche Sammlung von Mikroorganismen und Zellkulturen GmbH, Braunschweig, Germany). MOPS medium was prepared, gassed with N_2, and dispensed in an anaerobic chamber (Coy Laboratory Products, Ann Arbor, MI) under an atmosphere of 95% N_2 and 5% H_2. Cysteine-sulfide solution as a reducing agent was added to anaerobic medium (the final concentrations of L-cysteine · HCl and $Na_2S \cdot 9H_2O$ were 0.025%). WTE, an aqueous extract from *Acacia mearnsii* (black wattle) bark, was added to the MOPS medium at a final concentration of 1% (wt/vol). WTE contained 65.6% phenols as tannic acid equivalents and was donated by Wickett and Craig of America, Inc. (Curwensville, PA). WTE was filter sterilized before addition to growth medium. In order to identify specific transcriptional responses to the presence of tannins, as opposed to differences in environmental conditions and growth rate, *E. coli* BW13711 cells were grown anaerobically in continuous culture (dilution rate = 0.14 h^{-1}) in the presence and absence of WTE at 37°C. Under both steady-state conditions, *E. coli* reached an optical density at 600 nm (OD_{600}) of ~0.2. The pH values at steady state were 5.7 and 5.3 without and with WTE, respectively. These conditions indicated that the presence of tannins in the medium did not inhibit the growth of *E. coli*. *E. coli* BW13711 cells were harvested from continuous culture after steady-state conditions were reached. Duplicate samples were collected after an additional 4-volume turnover of the culture medium, and total RNA was isolated from these samples by using an RNeasy mini kit (Qiagen Inc., Valencia, CA). The presence of residual DNA was checked by PCR using the primers for amplification of *cca* (see Table S2 in the supplemental material). When necessary, the remaining DNA was removed by RQ1 RNase-free DNase (Promega Corp., Madison, WI) according to the manufacturer's instructions. *E. coli* whole-genome transcriptional profiling was performed by hybridization on the GeneChip *E. coli* antisense genome array (Affymetrix Inc., Santa Clara, CA) according to the manufacturer's instructions. Microarray data were analyzed using MicroArray Suite 5.0 (Affymetrix Inc.) and GeneSpring 5.0 (Silicon Genetics, Redwood City, CA) software. The 95% confidence interval of the WTE array duplicates demonstrated that genes >1.9-fold up- or down-regulated could be considered significantly differentially regulated. After removal of significantly up- or down-regulated genes which had large variability in expression on the duplicate arrays, it was found that in total 1,498 genes were up- or down-regulated (see Table S1 in the supplemental material). Genes from a wide variety of functional

* Corresponding author. Mailing address: Agtech Products, Inc., W227 N752 Westmound Dr., Waukesha, WI 53186. Phone: (262) 521-1717. Fax: (262) 521-2442. E-mail: xsmith@agtechproducts.com.

† Supplemental material for this article may be found at http://aem.asm.org/.

‡ Current address: Laboratory of Microbiology, Wageningen University, Dreijenplein 10, 6703 HB Wageningen, The Netherlands.

▽ Published ahead of print on 26 November 2007.

TABLE 1. *E. coli* strains used in this study

Strain	Genotype	Source or reference
BW13711[a]	Δ(*lac*)X74 17	8
WTT1	Spontaneous wattle tannin-tolerant mutant of BW13711	19
ΔBaeSR13711	ΔBaeSR mutant of BW13711	This study
BW25113[b]	*lacIp4000*(*lacI*^{q}) *rrnB3* Δ*lacZ4787* *hsdR514* Δ(*araBAD*)*567* Δ (*rhaBAD*)*568* *rph-1*	4
BW27553[b]	ΔBaeSR mutant of BW25113	26
BW28357[b]	*rph*$^+$ derivative of BW25113	4
BW29744[b]	ΔBaeSR mutant of BW28357	26

[a] Supplied by W. Metcalf, University of Illinois, Urbana, IL.
[b] Supplied by *E. coli* Genetic Stock Center, New Haven, CT.

classes were regulated, with "viral functions," "protein fate," and "unclassified" as functional categories with the highest percentages of up-regulated genes (Fig. 1). On the other hand, "protein synthesis," "amino acids," and "nucleic acid building blocks" were the categories with the highest percentages of down-regulated genes.

Gene expression responses determined with the Affymetrix GeneChip were verified with quantitative reverse transcription-PCR (qRT-PCR). Primers were developed for amplifying mRNA from 28 single genes, which included 2 constitutive control genes, *cca* and *dnaC* (see Table S2 in the supplemental material). Neither control gene was differentially expressed in any of the experiments reported here, based on Affymetrix GeneChip data. Besides the constitutive control genes, the 10 most highly up-regulated genes, supplemented with additional, randomly selected genes, including the most down-regulated gene, were selected for gene-specific qRT-PCR. In addition, the *mdtABCD* and *marRAB* operon genes were included, as will be explained later (see Table S1 in the supplemental material). After the PCR conditions were optimized, the suitability of the primers for qRT-PCR was determined by using total RNA isolated from *E. coli* grown in WTE medium in a 10-fold dilution series (from 10^0 to 10^{-4}) as templates. These RNA dilution series were subsequently used as standards for qRT-PCR of specific gene expression. qRT-PCR was performed on a GeneAmp 5700 sequence detection system, using a Quantitect SYBR green RT-PCR kit (Qiagen Inc., Valencia, CA). A total volume of 25 μl per sample consisted of 8.75 μl of RNase-free water, 12.5 μl of 2× Quant SYBR green RT-PCR master mix, 1.25 μl of each primer (10 μM), 0.25 μl of Quant RT mix, and 1.0 μl of (diluted) RNA. A one-step real-time RT-PCR protocol was performed as follows: warming up at 50°C for 2 min, RT at 50°C for 30 min, PCR initiation at 94°C for 15 min, and 40 amplification cycles (94°C, 15 s; 60°C, 30 s; and 72°C, 30 s). These conditions were used for all primer pairs (see Table S2 in the supplemental material), with the exception of those amplifying *ylbE*, for which 45 cycles of amplification and 10-fold-diluted primer concentrations were used to minimize primer-dimer formation. The gene expression ratios (Table 2) were calculated after normalization of the data by use of the expression ratios from the two constitutive control genes *cca* and *dnaC*.

Contrary to expectations, genes involved in energy metabolism were up-regulated in WTE medium. For example, the

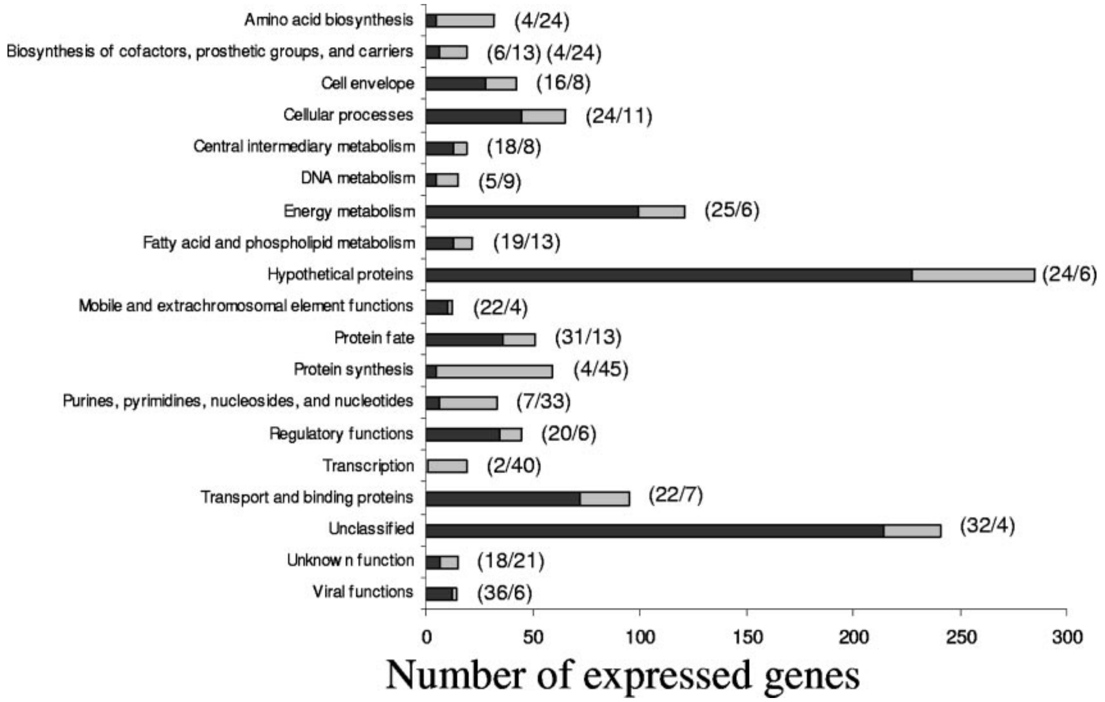

FIG. 1. Representation of *E. coli* BW13711 genes that are regulated during exposure to black wattle tannins. The genes are categorized according to the TIGR functional annotation (http://cmr.tigr.org/). Genes for which no categorization data were found were excluded. Up- and down-regulated genes are represented in black and gray, respectively. The percentages of up- and down-regulated genes compared to the total number of genes in each functional category are indicated in brackets.

TABLE 2. Relative expression levels of *Escherichia coli* genes grown in a 1% WTE-containing medium as determined by Affymetrix microarray and qRT-PCR analyses

Locus tag	Gene[a]	Fold change		Gene function (cellular role[s])[b]
		Microarray	qRT-PCR	
b1743	*spy*[c]	17.5	493.5	Periplasmic protein related to spheroblast formation (unclassified)
b2074	*mdtA*[c]	4.5	35.9	Multidrug efflux system, subunit A (cellular processes; toxin production and resistance)
b2075	*mdtB*	4.4	24.8	Multidrug efflux system, subunit B (cellular processes; toxin production and resistance)
b2076	*mdtC*	2.1	15.3	Multidrug efflux system, subunit C (cellular processes; toxin production and resistance)
b2077	*mdtD*	3.8	27.6	Multidrug efflux system, subunit D (cellular processes; toxin production and resistance)
b1531	*marA*	6.3	22.5	Multiple antibiotic resistance; transcriptional activator of defense systems (cellular processes; toxin production and resistance)
b1532	*marB*	2.6	21.5	Multiple antibiotic resistance protein (cellular processes; toxin production and resistance)
b1530	*marR*	2.6	27.4	DNA-binding transcriptional repressor of multiple antibiotic resistance (cellular processes; toxin production and resistance)
b0005	*yaaX*	4.9	26.6	Hypothetical protein (unclassified)
b2390	*ypeC*	2.8	20.5	Hypothetical protein (unclassified)
b0618	*citC*	34.6	14.7	Citrate lyase synthetase (energy metabolism; TCA cycle)
b3687	*ibpA*	6.9	10.4	Heat shock chaperone (protein fate; protein folding and stabilization)
b3686	*ibpB*	9.5	48.0	Heat shock chaperone (protein fate; protein folding and stabilization)
b3238	*yhcN*	3.1	7.9	Hypothetical protein (unclassified)
b2675	*nrdE*	26.8	5.2	Ribonucleoside-diphosphate reductase (purines, pyrimidines, nucleosides, and nucleotides; 2′-deoxyribonucleotide metabolism)
b3119	*tdcR*	13.1	4.7	Threonine dehydratase operon activator protein (unclassified)
b1306	*pspC*	3.6	4.6	Phage shock protein: activates phage shock-protein expression (unclassified)
b3265	*acrE*	12.1	3.1	Membrane fusion protein (cell envelope; biosynthesis and degradation of murein sacculus and peptidoglycan)
b3071	*yqjI*	2.9	2.1	Predicted transcriptional regulator (unclassified)
b0929	*ompF*	0.1	0.04	Outer membrane protein 1a (transport and binding proteins; porins)
b3056[d]	*cca*	1.0	1.0	tRNA nucleotidyl transferase (protein synthesis tRNA and rRNA base modification)
b4361[d]	*dnaC*	1.1	0.9	DNA biosynthesis protein (viral functions; general)
b2871[e]	*ygeX*	27.1	0.9	Putative diaminopropionate ammonia-lyase (energy metabolism; amino acids and amines)
b2971[e]	*yghG*	17.5	0.4	Hypothetical protein (unclassified)
b3948[e]	*yijI*	28.6	1.4	Hypothetical protein (NCBI record discontinued)
b0303[e]	*ykgI*	13.2	0.4	Hypothetical protein (unclassified)
b0519[e]	*ylbE*	26.2	1.1	Pseudogene (unclassified)

[a] Based on information from the NCBI genome resources (http://www.ncbi.nlm.nih.gov/genomes/lproks.cgi).
[b] Based on information from TIGR Cellular Role Category (http://cmr.tigr.org/tigr-scripts/CMR/CmrHomePage.cgi).
[c] BaeR consensus binding region upstream of the gene.
[d] Constitutively expressed control genes.
[e] Genes for which there was no correlation between the microarray and qRT-PCR data.

expression of *citC*, an activator of citrate lyase, was increased 14.7-fold, as determined by qRT-PCR, in WTE medium. The expression levels of metabolic genes and the lower pH in the presence of WTE indicated that additional carbon sources were available for *E. coli* in WTE. *E. coli* BW13711 was therefore grown with WTE as the sole C source. The growth rate of *E. coli* BW13711 on WTE medium without glucose was similar to that on WTE medium and MOPS medium with glucose, but the yield was lower (end OD_{600}, ~0.2 instead of ~0.6) (data not shown). This could indicate that other, possibly sugar-containing compounds were coextracted in the crude WTE. Even though there appeared to be an increase in energy metabolism, the presence of WTE caused apparent down-regulation of genes involved in the biosynthesis of amino acids, proteins, nucleotides, and cofactors as well as transcription (Fig. 1). It remains to be investigated whether the down-regulation of these biosynthetic pathways is related to an alteration in the physiological state of the cells in the presence of tannins or to the presence of additional compounds that are coextracts in the WTE.

The expression of *spy* was increased 493.5-fold, as determined by qRT-PCR, in WTE medium. The exact function of Spy (*s*pheroblast *p*rotein *y*) (8) is unknown, but it is suggested to be involved in outer membrane protein folding and cell envelope synthesis (15). *spy* is regulated by the BaeSR two-component system, which was found to be important in the tolerance of *E. coli* toward gallic acid, a simple phenolic compound, under aerobic conditions (26). This BaeSR two-component system has also recently been described to regulate the *mdtABCD* operon, which encodes a recently identified multidrug transport system (2, 11, 14). Indeed, this operon was also found to be significantly up-regulated, as determined by gene-specific qRT-PCR (Table 2). To verify the importance of BaeSR in the resistance of *E. coli* toward tannins, ΔBaeSR knockout mutants were constructed using amplicons for chromosomal gene disruptions with the phage λ Red recombinase one-step gene inactivation protocol (4). A direct construction of this mutant was not possible, which is likely due to the fact that *E. coli* BW13711 can utilize arabinose, the inducer of the λ Red recombinase activity, thus hampering the gene disruption efficiency. Therefore, the Δ*baeSR*::*kan* transformant was first constructed in *E. coli* BW25113 by using primers *baeS*-H1-P1 and *baeR*-H2-P4 (see Table S3 in the supplemental material), since successful construction of a BaeSR knockout was previously demonstrated for this strain (26). The resulting mutant was used as a template to construct *E. coli*

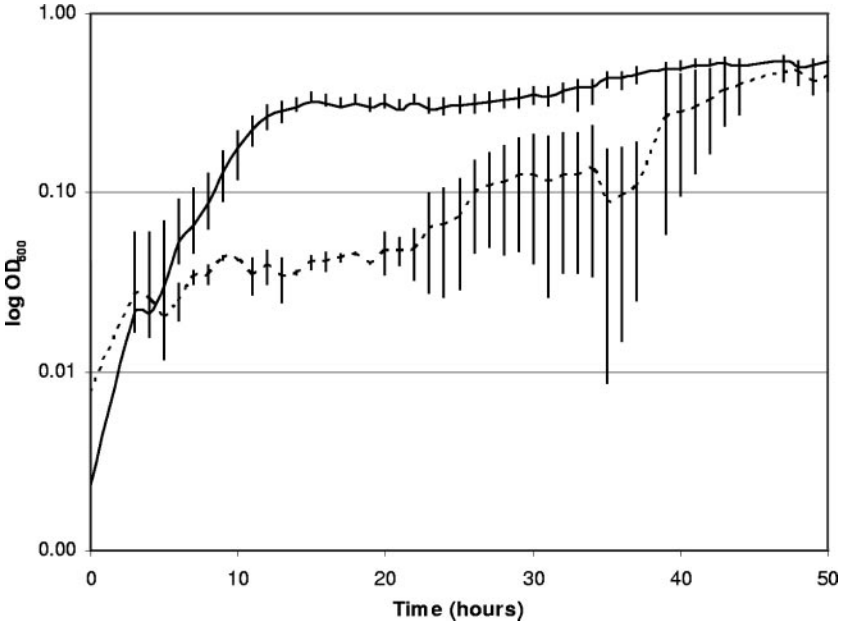

FIG. 2. Growth curves of *E. coli* BW13711 (solid line) and the *baeSR* deletion mutant ΔBaeSR13711 (dashed line) inoculated in MOPS medium containing 1% WTE. The means and standard deviations for six replicates are indicated.

ΔBaeSR13711 by using the *yeg*P-R primer and the *mdtD* forward primer, since this generated amplicons with longer regions of homology to the chromosome. Wild-type and mutant strains of *E. coli* were grown in anaerobic batch culture on MOPS medium with and without 1.0% WTE. Growth curves were determined by measuring the OD_{600} values at regular time intervals.

The growth rates in standard MOPS medium with glucose were similar for *E. coli* strains BW13711, ΔBaeSR13711, and WTT1 (a spontaneous mutant that is not sensitive to tannins under oxic conditions) (Table 1), indicating that the mutants have not lost additional functions needed for anaerobic growth in this medium. When WTE was present in MOPS medium, the growth rates of strains BW13711 and WTT1 were identical (data not shown). There was a significant lag time of approximately 35 h before strain ΔBaeSR13711 grew on medium containing WTE (Fig. 2). Results were similar for two other wild-type strains of *E. coli* (BW25113 and BW28357) and their ΔBaeSR mutants (BW27553 and BW29744, respectively) (data not shown). Interestingly, the lag times of the mutants were reduced to levels close to those of the respective wild-type strains when WTE medium was stored at 37°C for 40 h prior to incubation (data not shown). This suggests that components in the media, possibly the metallic trace elements, reacted with the hydroxyl groups of the tannins and thereby decreased the tannic reactivity. This loss of tannin reactivity can explain the sudden growth of the ΔBaeSR mutants after 35 h, whereas the wild-type strains had reached stationary phase by 16 h.

It is evident that the BaeSR two-component system has a prominent role in the mechanisms for the resistance of *E. coli* toward the presence of tannins. Interestingly, a similar two-component system in *Pseudomonas putida* has been described. In this case, the ColRS two-component system protected *P. putida* against phenol by regulating membrane functionality (7).

A recent paper indicated that BaeSR is part of a cross-regulation system which includes the PhoBR and CreBC two-component systems (13). This suggests that constructing ΔBaeSR mutants may not just affect the expression levels of genes that are under the direct control of BaeSR but may even cause disruptions in a complex regulatory network. The impact of tannins on this cross-regulation among BaeSR, PhoBR, and CreBC remains to be addressed in future studies.

The most down-regulated gene was *ompF*, encoding an outer membrane porin for small organic molecules (3). The down-regulation of this gene has been described to be regulated by the *marRAB* (*m*ultiple-*a*ntibiotic-*r*esistance) regulon. This regulon was also found to be significantly up-regulated in WTE medium, as determined by gene-specific qRT-PCR (Table 2). Interestingly, this operon is expressed under various stress conditions, including the presence of phenolic compounds (24).

The finding that low-level efflux pump antibiotic resistance mechanisms (*marRAB* and *mdtABCD* operons) are up-regulated in the presence of naturally occurring plant secondary compounds is intriguing. If further studies confirm that these efflux pumps are integral to the resistance of *E. coli* to tannins, then the potential implication is that plant secondary compounds that are well documented in terms of biological activity, such as the oligomers of flavanols (proanthocyanidins or condensed tannins in *A. mearnsii*), also provide a nonantibiotic selection mechanism for development, maintenance, and transfer of antibiotic resistance. Antibiotic selective pressure is not required for the selection of antibiotic resistance genes carried by replicons that contain other selectable markers, such as heavy metal resistance genes (1, 5, 25). The gastrointestinal

tract is a natural arena for such a mechanism to operate since it combines a large reservoir of commensal bacteria of high density and wide genomic diversity with a daily intake of dietary material containing fruit and fruit juices, tea, wine, vegetables, and fiber, which are natural sources of plant secondary compounds, such as flavanols. Our hypothesis is that this may, in part, account for the "easy-to-gain" and "hard-to-lose" resistance gene phenomenon (16), but more extensive and rigorous experimentation is required in order to study these resistance mechanisms in commensal gastrointestinal bacteria and the role of flavanols in the development, selection, and maintenance of antibiotic resistance.

In conclusion, *E. coli* overcomes the inhibitory effect of tannins by a variety of mechanisms, which include genes that are under the control of the BaeSR two-component system, such as the *mdtABCD* efflux pump gene and the outer membrane protein gene *spy*, in WTE medium. In addition, the downregulation of the outer membrane porin gene *ompF*, mediated by the *marRAB* regulon, seems to be important in this resistance. We hypothesize that these systems prevent cytosolic and membrane damage caused by condensed tannin fractions. While this study has provided insight into the mechanisms involved in tannin resistance in *E. coli*, this resistance appeared to be very complex, leaving many questions that remain to be answered.

Microarray data accession number. Microarray data have been deposited in the Gene Expression Omnibus database (http://www.ncbi.nlm.nih.gov/) under accession number GSE9755.

D. H. Akan from the W. M. Keck Center for Comparative and Functional Genomics is acknowledged for his technical support of the DNA microarray analysis. L. Liu and S. Rodrigues-Zas are acknowledged for their advice in analyzing the mass of DNA microarray data. We thank W. Metcalf and B. Wanner for providing the *E. coli* strains. We also thank W. Metcalf from the Dept. of Microbiology for helpful advice and discussion concerning the construction of the ΔBaeSR mutants and Michiel Wels from the Centre for Molecular and Biomolecular Informatics at the Radboud University Nijmegen-Medical Centre/NCMLS for his help in categorizing the genes into functional groups. Hauke Smidt from the Laboratory of Microbiology at Wageningen University is acknowledged for critically reading the manuscript.

This work was sponsored by the Illinois Council for Food and Agricultural Research (C-FAR) (E.G.Z.). Partial support was also provided by the International Arid Lands Consortium (A.H.S.), the Norwegian Research Council (M.A.S.), USDA National Research Initiative 26.0 (proposal no. 2005-35102-16426) (R.I.M.), and the Agricultural Experiment Station of the University of Illinois.

REFERENCES

1. **Alonso, A., P. Sanchez, and J. L. Martinez.** 2001. Environmental selection of antibiotic resistance genes. Environ. Microbiol. **3:**1–9.
2. **Baranova, N., and H. Nikaido.** 2002. The *baeSR* two-component regulatory system activates transcription of the *yegMNOB* (*mdtABCD*) transporter gene cluster in *Escherichia coli* and increases its resistance to novobiocin and deoxycholate. J. Bacteriol. **184:**4168–4176.
3. **Barbosa, T. M., and S. B. Levy.** 2000. Differential expression of over 60 chromosomal genes in *Escherichia coli* by constitutive expression of MarA. J. Bacteriol. **182:**3467–3474.
4. **Datsenko, K. A., and B. L. Wanner.** 2000. One-step inactivation of chromosomal genes in *Escherichia coli* K-12 using PCR products. Proc. Natl. Acad. Sci. USA **97:**6640–6645.
5. **Davison, J.** 1999. Genetic exchange between bacteria in the environment. Plasmid **42:**73–91.
6. **Jones, G. A., T. A. McAllister, A. D. Muir, and K. J. Cheng.** 1994. Effects of sainfoin (*Onobrychis viciifolia* Scop.) condensed tannins on growth and proteolysis by four strains of ruminal bacteria. Appl. Environ. Microbiol. **60:** 1374–1378.
7. **Kivistik, P. A., M. Putrins, K. Puvi, H. Ilves, M. Kivisaar, and R. Horak.** 2006. The ColRS two-component system regulates membrane functions and protects *Pseudomonas putida* against phenol. J. Bacteriol. **188:**8109–8117.
8. **Metcalf, W. W., P. M. Steed, and B. L. Wanner.** 1990. Identification of phosphate starvation-inducible genes in *Escherichia coli* K-12 by DNA sequence analysis of *psi*::*lacZ*(Mu d1) transcriptional fusions. J. Bacteriol. **172:**3191–3200.
9. **Min, B. R., G. T. Attwood, K. Reilly, W. Sun, J. S. Peters, T. N. Barry, and W. C. McNabb.** 2002. *Lotus corniculatus* condensed tannins decrease in vivo populations of proteolytic bacteria and affect nitrogen metabolism in the rumen of sheep. Can. J. Microbiol. **48:**911–921.
10. **Molan, A. L., G. T. Attwood, B. R. Min, and W. C. McNabb.** 2001. The effect of condensed tannins from *Lotus pedunculatus* and *Lotus corniculatus* on the growth of proteolytic rumen bacteria in vitro and their possible mode of action. Can. J. Microbiol. **47:**626–633.
11. **Nagakubo, S., K. Nishino, T. Hirata, and A. Yamaguchi.** 2002. The putative response regulator BaeR stimulates multidrug resistance of *Escherichia coli* via a novel multidrug exporter system, MdtABC. J. Bacteriol. **184:**4161–4167.
12. **Neidhardt, F. C., P. L. Bloch, and D. F. Smith.** 1974. Culture medium for *Enterobacteria*. J. Bacteriol. **119:**736–747.
13. **Nishino, K., T. Honda, and A. Yamaguchi.** 2005. Genome-wide analyses of *Escherichia coli* gene expression responsive to the BaeSR two-component regulatory system. J. Bacteriol. **187:**1763–1772.
14. **Raffa, R. G., and T. L. Raivio.** 2002. A third envelope stress signal transduction pathway in *Escherichia coli*. Mol. Microbiol. **45:**1599–1611.
15. **Raivio, T. L., M. W. Laird, J. C. Joly, and T. J. Silhavy.** 2000. Tethering of CpxP to the inner membrane prevents spheroplast induction of the *cpx* envelope stress response. Mol. Microbiol. **37:**1186–1197.
16. **Salyers, A. A., and C. F. Amabile-Cuevas.** 1997. Why are antibiotic resistance genes so resistant to elimination? Antimicrob. Agents Chemother. **41:**2321–2325.
17. **Schofield, P., D. M. Mbugua, and A. N. Pell.** 2001. Analysis of condensed tannins: a review. Anim. Feed Sci. Technol. **91:**21–40.
18. **Silanikove, N., N. Gilboa, and Z. Nitsan.** 2001. Effect of polyethylene glycol on rumen volume and retention time of liquid and particulate matter along the digestive tract in goats fed tannin-rich carob leaves (*Ceratonia siliqua*). Small Rumin. Res. **40:**95–99.
19. **Smith, A. H., J. A. Imlay, and R. I. Mackie.** 2003. Increasing the oxidative stress response allows *Escherichia coli* to overcome inhibitory effects of condensed tannins. Appl. Environ. Microbiol. **69:**3406–3411.
20. **Smith, A. H., and R. I. Mackie.** 2004. Effect of condensed tannins on bacterial diversity and metabolic activity in the rat gastrointestinal tract. Appl. Environ. Microbiol. **70:**1104–1115.
21. **Smith, A. H., A. A. Odenyo, P. O. Osuji, M. A. Wallig, F. E. Kandil, D. S. Seigler, and R. I. Mackie.** 2001. Evaluation of toxicity of *Acacia angustissima* in a rat bioassay. Anim. Feed Sci. Technol. **91:**41–57.
22. **Smith, A. H., E. Zoetendal, and R. I. Mackie.** 2005. Bacterial mechanisms to overcome inhibitory effects of dietary tannins. Microb. Ecol. **50:**197–205.
23. **Sreeangaraju, G., U. Krishnamoorthy, and M. M. Kailas.** 2000. Evaluation of Bengal gram (*Cicer arietinum*) husk a source of tannin and its interference in rumen and post-rumen nutrient digestion in sheep. Anim. Feed Sci. Technol. **85:**131–138.
24. **Sulavik, M. C., L. F. Gambino, and P. F. Miller.** 1995. The MarR repressor of the multiple antibiotic resistance (*mar*) operon in *Escherichia coli*: prototypic member of a family of bacterial regulatory proteins involved in sensing phenolic compounds. Mol. Med. **1:**436–446.
25. **Summers, A. O., J. Wireman, M. J. Vimy, F. L. Lorscheider, B. Marshall, S. B. Levy, S. Bennett, and L. Billard.** 1993. Mercury released from dental "silver" fillings provokes an increase in mercury- and antibiotic-resistant bacteria in oral and intestinal floras of primates. Antimicrob. Agents Chemother. **37:**825–834.
26. **Zhou, L., X. H. Lei, B. R. Bochner, and B. L. Wanner.** 2003. Phenotype microarray analysis of *Escherichia coli* K-12 mutants with deletions of all two-component systems. J. Bacteriol. **185:**4956–4972.
