# Supplementary figures and images for: MetNetGE: interactive views of biological networks and ontologies
Source: BMC Bioinformatics. 2010 Sep 17;11:469. doi: 10.1186/1471-2105-11-469 (PMC2946353; doi:10.1186/1471-2105-11-469)

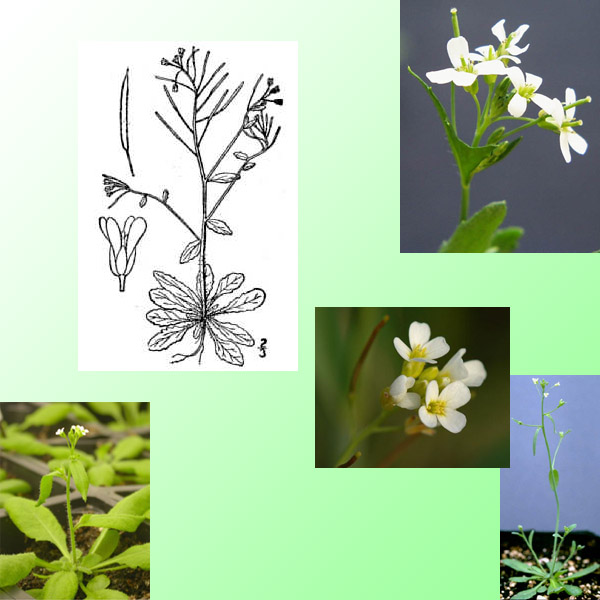

Supplement: Additional file 2 — MetNetGE source code. This is the source code of MetNetGE. Please go to MetNetGE.org to find the latest version, dependency packages and tutorials. [file 1471-2105-11-469-S2.ZIP › MetNetGE-1.0.3/NetworkGE/Sample/basedata/files/ArabiAll.jpg]

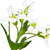

Supplement: Additional file 2 — MetNetGE source code. This is the source code of MetNetGE. Please go to MetNetGE.org to find the latest version, dependency packages and tutorials. [file 1471-2105-11-469-S2.ZIP › MetNetGE-1.0.3/NetworkGE/Sample/basedata/files/ArabiIcon.png]

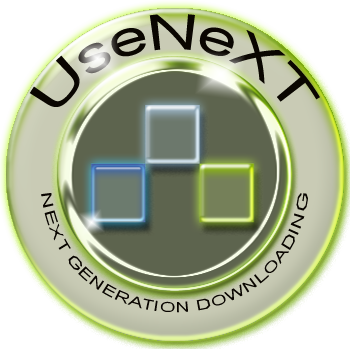

Supplement: Additional file 2 — MetNetGE source code. This is the source code of MetNetGE. Please go to MetNetGE.org to find the latest version, dependency packages and tutorials. [file 1471-2105-11-469-S2.ZIP › MetNetGE-1.0.3/NetworkGE/Sample/basedata/files/base.png]

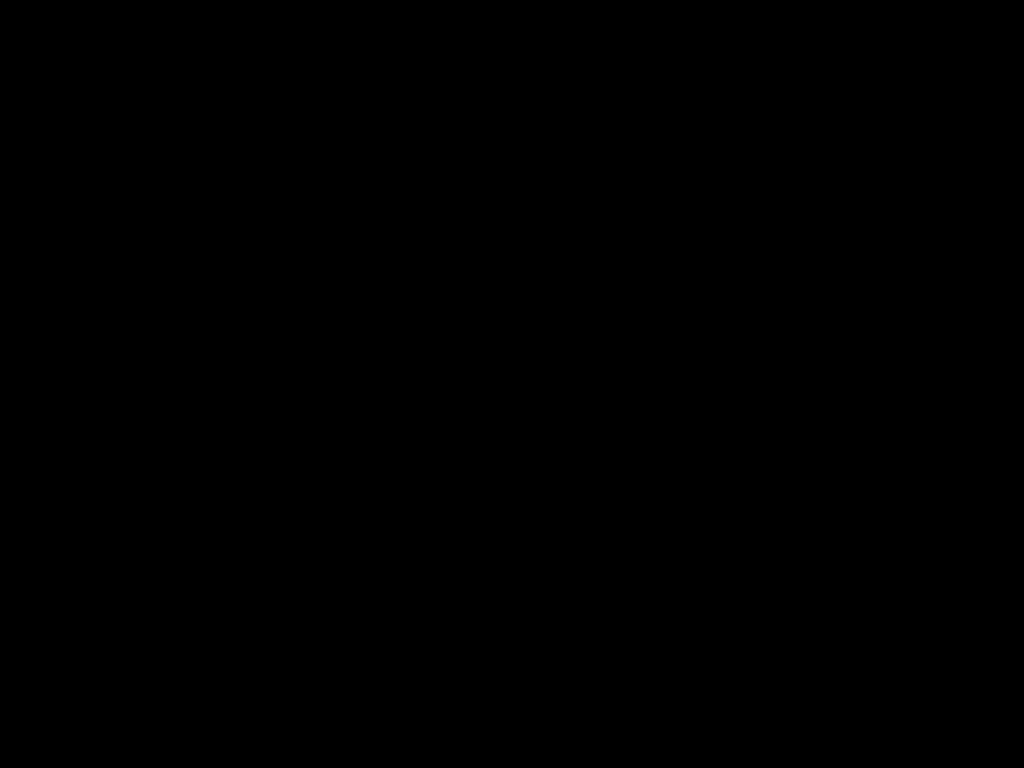

Supplement: Additional file 2 — MetNetGE source code. This is the source code of MetNetGE. Please go to MetNetGE.org to find the latest version, dependency packages and tutorials. [file 1471-2105-11-469-S2.ZIP › MetNetGE-1.0.3/NetworkGE/Sample/basedata/files/Black.jpg]

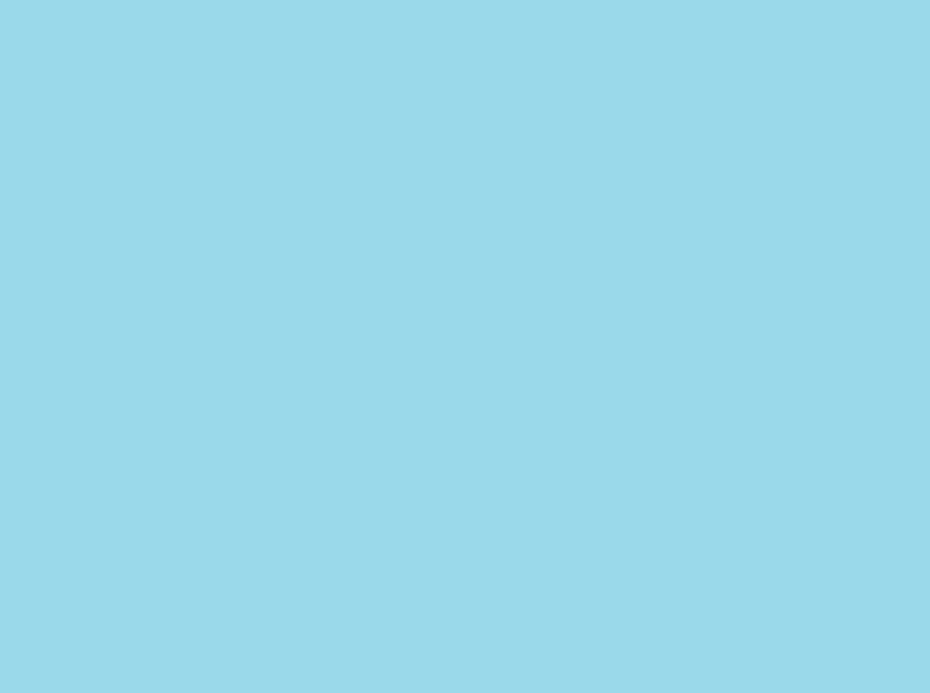

Supplement: Additional file 2 — MetNetGE source code. This is the source code of MetNetGE. Please go to MetNetGE.org to find the latest version, dependency packages and tutorials. [file 1471-2105-11-469-S2.ZIP › MetNetGE-1.0.3/NetworkGE/Sample/basedata/files/Blue.jpg]

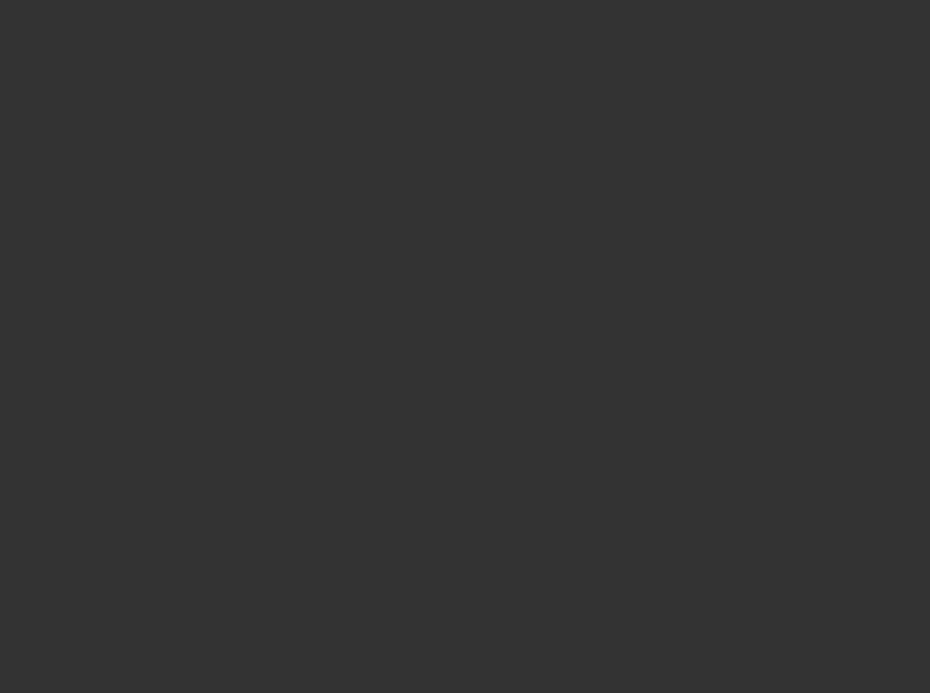

Supplement: Additional file 2 — MetNetGE source code. This is the source code of MetNetGE. Please go to MetNetGE.org to find the latest version, dependency packages and tutorials. [file 1471-2105-11-469-S2.ZIP › MetNetGE-1.0.3/NetworkGE/Sample/basedata/files/DarkGray.jpg]

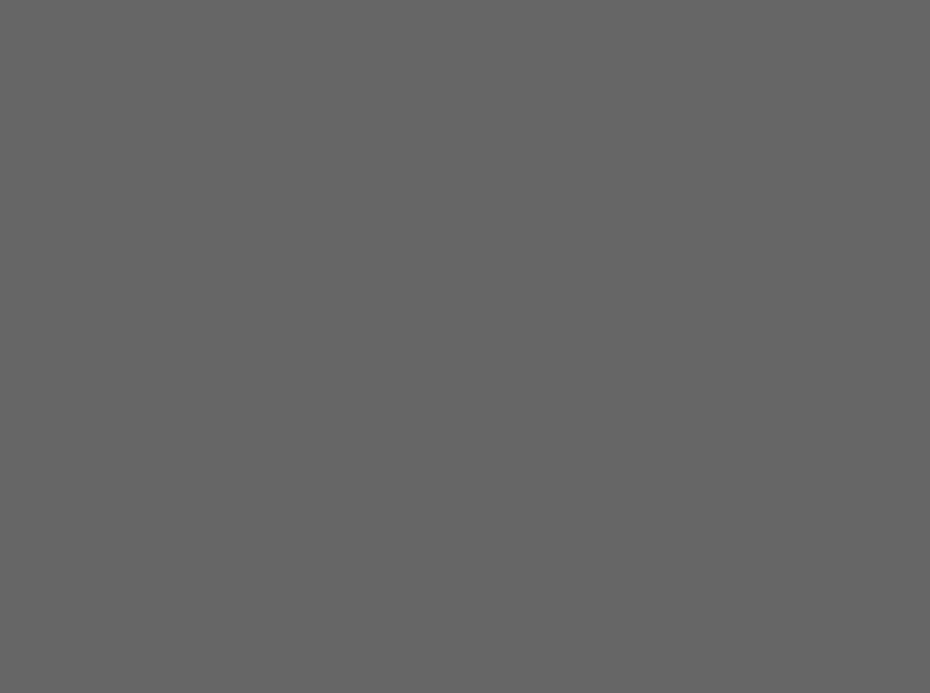

Supplement: Additional file 2 — MetNetGE source code. This is the source code of MetNetGE. Please go to MetNetGE.org to find the latest version, dependency packages and tutorials. [file 1471-2105-11-469-S2.ZIP › MetNetGE-1.0.3/NetworkGE/Sample/basedata/files/DarkGray2.jpg]

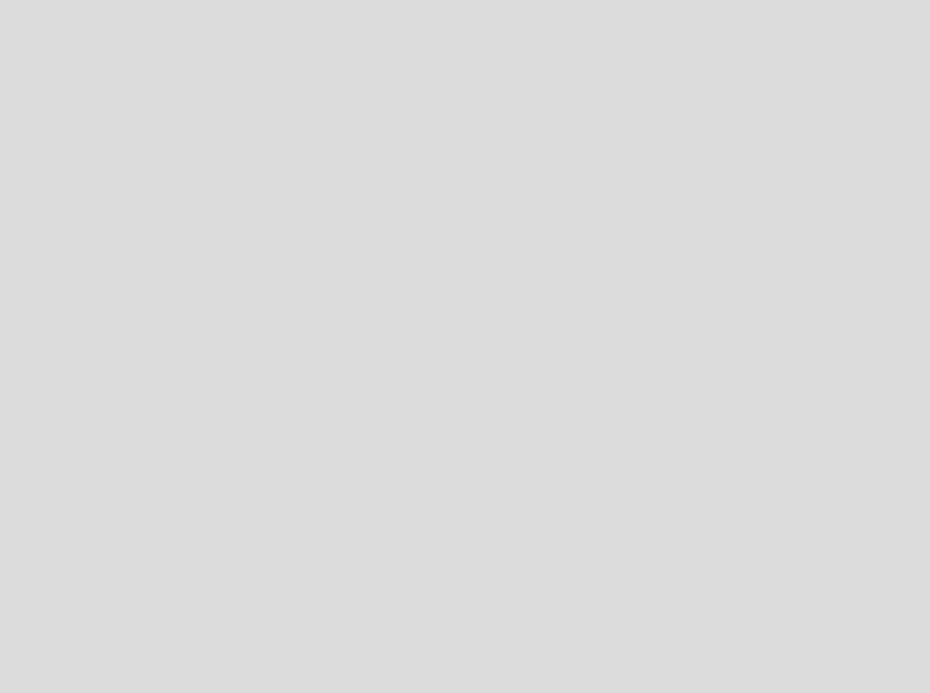

Supplement: Additional file 2 — MetNetGE source code. This is the source code of MetNetGE. Please go to MetNetGE.org to find the latest version, dependency packages and tutorials. [file 1471-2105-11-469-S2.ZIP › MetNetGE-1.0.3/NetworkGE/Sample/basedata/files/Gray.jpg]

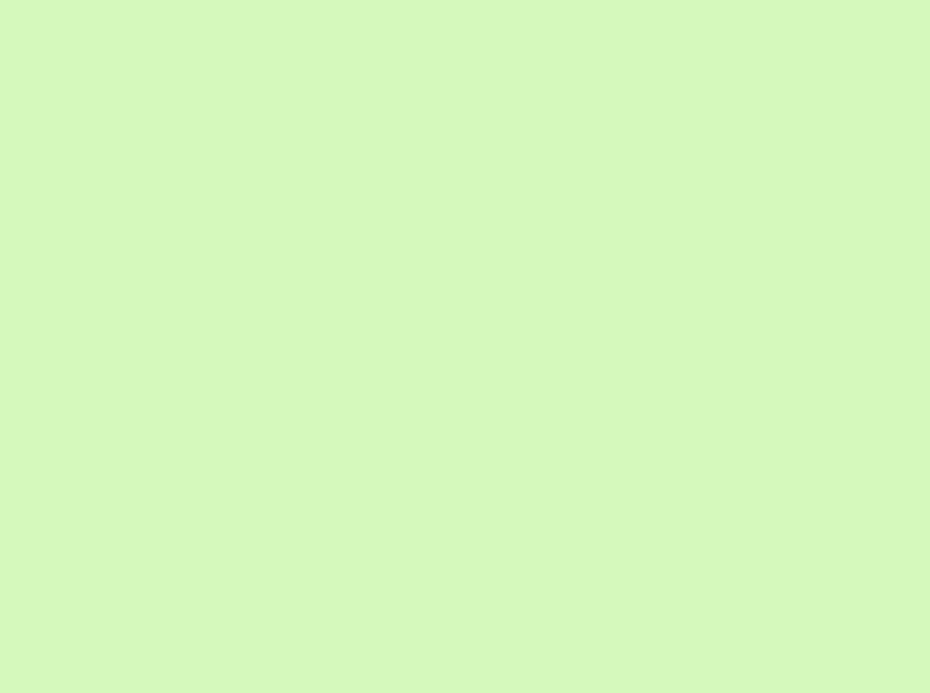

Supplement: Additional file 2 — MetNetGE source code. This is the source code of MetNetGE. Please go to MetNetGE.org to find the latest version, dependency packages and tutorials. [file 1471-2105-11-469-S2.ZIP › MetNetGE-1.0.3/NetworkGE/Sample/basedata/files/Green.jpg]

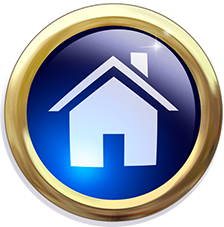

Supplement: Additional file 2 — MetNetGE source code. This is the source code of MetNetGE. Please go to MetNetGE.org to find the latest version, dependency packages and tutorials. [file 1471-2105-11-469-S2.ZIP › MetNetGE-1.0.3/NetworkGE/Sample/basedata/files/HomeIcon1.png]

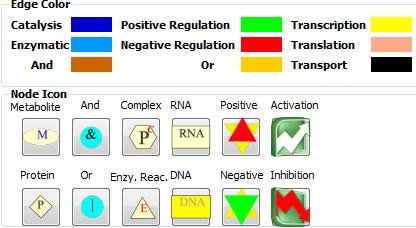

Supplement: Additional file 2 — MetNetGE source code. This is the source code of MetNetGE. Please go to MetNetGE.org to find the latest version, dependency packages and tutorials. [file 1471-2105-11-469-S2.ZIP › MetNetGE-1.0.3/NetworkGE/Sample/basedata/files/Legend.jpg]

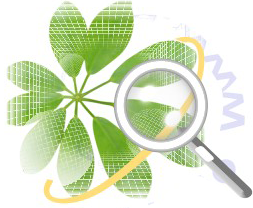

Supplement: Additional file 2 — MetNetGE source code. This is the source code of MetNetGE. Please go to MetNetGE.org to find the latest version, dependency packages and tutorials. [file 1471-2105-11-469-S2.ZIP › MetNetGE-1.0.3/NetworkGE/Sample/basedata/files/networkge_icon1.png]

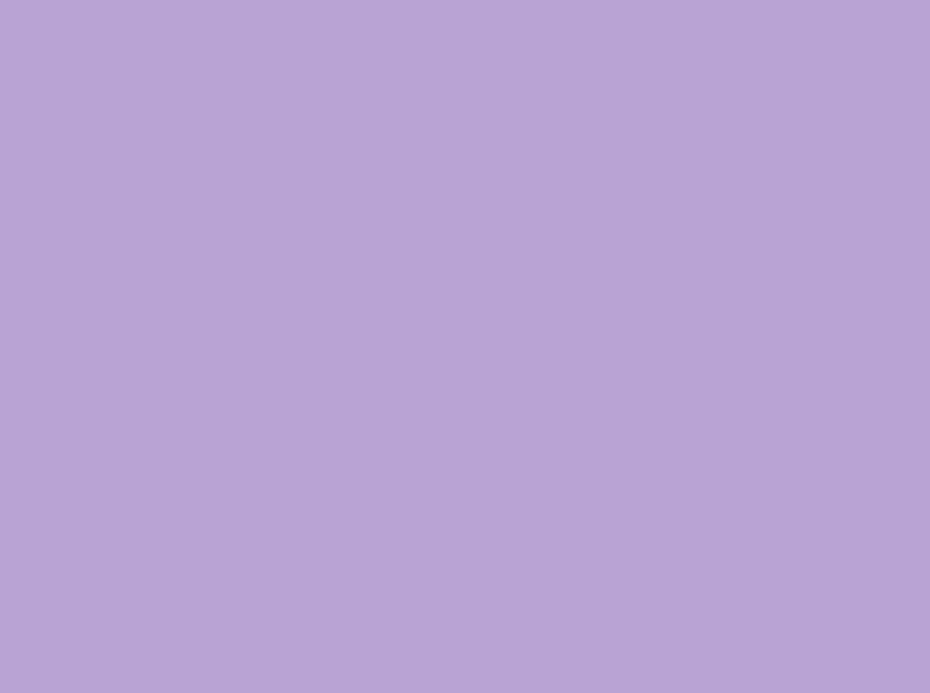

Supplement: Additional file 2 — MetNetGE source code. This is the source code of MetNetGE. Please go to MetNetGE.org to find the latest version, dependency packages and tutorials. [file 1471-2105-11-469-S2.ZIP › MetNetGE-1.0.3/NetworkGE/Sample/basedata/files/Purple.jpg]

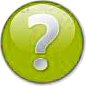

Supplement: Additional file 2 — MetNetGE source code. This is the source code of MetNetGE. Please go to MetNetGE.org to find the latest version, dependency packages and tutorials. [file 1471-2105-11-469-S2.ZIP › MetNetGE-1.0.3/NetworkGE/Sample/basedata/files/question2.png]

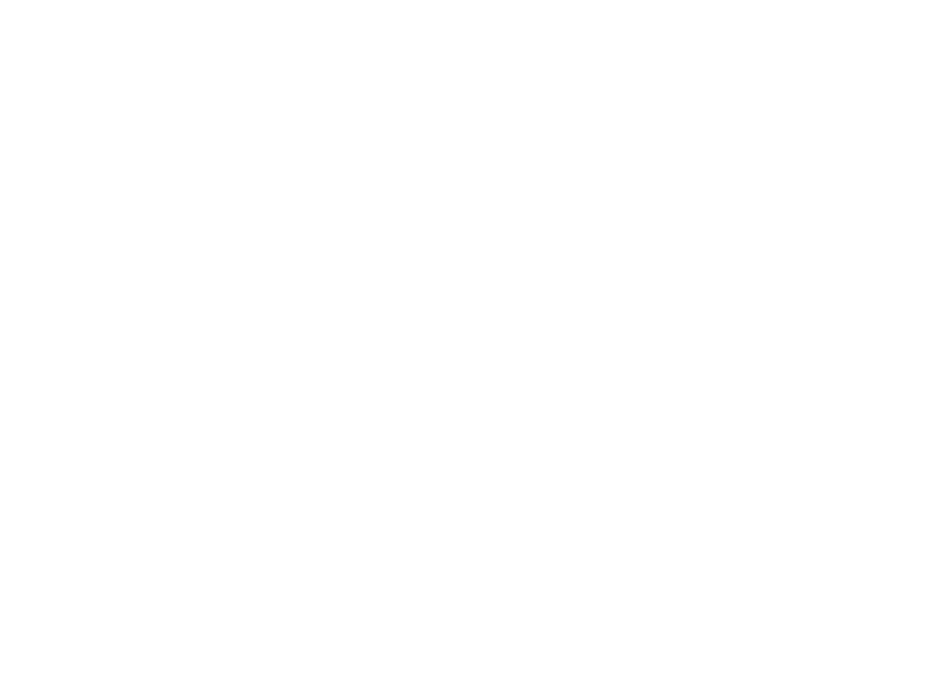

Supplement: Additional file 2 — MetNetGE source code. This is the source code of MetNetGE. Please go to MetNetGE.org to find the latest version, dependency packages and tutorials. [file 1471-2105-11-469-S2.ZIP › MetNetGE-1.0.3/NetworkGE/Sample/basedata/files/White.jpg]

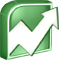

Supplement: Additional file 2 — MetNetGE source code. This is the source code of MetNetGE. Please go to MetNetGE.org to find the latest version, dependency packages and tutorials. [file 1471-2105-11-469-S2.ZIP › MetNetGE-1.0.3/NetworkGE/Sample/basedata/files/FCM/Activation.png]

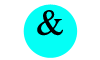

Supplement: Additional file 2 — MetNetGE source code. This is the source code of MetNetGE. Please go to MetNetGE.org to find the latest version, dependency packages and tutorials. [file 1471-2105-11-469-S2.ZIP › MetNetGE-1.0.3/NetworkGE/Sample/basedata/files/FCM/and.png]

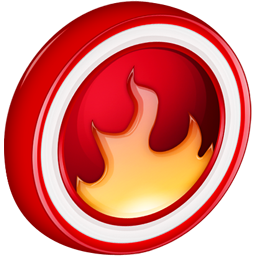

Supplement: Additional file 2 — MetNetGE source code. This is the source code of MetNetGE. Please go to MetNetGE.org to find the latest version, dependency packages and tutorials. [file 1471-2105-11-469-S2.ZIP › MetNetGE-1.0.3/NetworkGE/Sample/basedata/files/FCM/Catalysis.png]

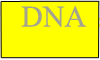

Supplement: Additional file 2 — MetNetGE source code. This is the source code of MetNetGE. Please go to MetNetGE.org to find the latest version, dependency packages and tutorials. [file 1471-2105-11-469-S2.ZIP › MetNetGE-1.0.3/NetworkGE/Sample/basedata/files/FCM/dna2.png]

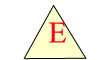

Supplement: Additional file 2 — MetNetGE source code. This is the source code of MetNetGE. Please go to MetNetGE.org to find the latest version, dependency packages and tutorials. [file 1471-2105-11-469-S2.ZIP › MetNetGE-1.0.3/NetworkGE/Sample/basedata/files/FCM/EnzymaticReaction2.png]

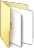

Supplement: Additional file 2 — MetNetGE source code. This is the source code of MetNetGE. Please go to MetNetGE.org to find the latest version, dependency packages and tutorials. [file 1471-2105-11-469-S2.ZIP › MetNetGE-1.0.3/NetworkGE/Sample/basedata/files/FCM/folder.png]

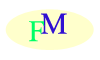

Supplement: Additional file 2 — MetNetGE source code. This is the source code of MetNetGE. Please go to MetNetGE.org to find the latest version, dependency packages and tutorials. [file 1471-2105-11-469-S2.ZIP › MetNetGE-1.0.3/NetworkGE/Sample/basedata/files/FCM/FrequentMetabolite.png]

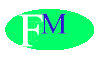

Supplement: Additional file 2 — MetNetGE source code. This is the source code of MetNetGE. Please go to MetNetGE.org to find the latest version, dependency packages and tutorials. [file 1471-2105-11-469-S2.ZIP › MetNetGE-1.0.3/NetworkGE/Sample/basedata/files/FCM/FrequentMetabolite2.png]

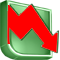

Supplement: Additional file 2 — MetNetGE source code. This is the source code of MetNetGE. Please go to MetNetGE.org to find the latest version, dependency packages and tutorials. [file 1471-2105-11-469-S2.ZIP › MetNetGE-1.0.3/NetworkGE/Sample/basedata/files/FCM/Inhibition.png]

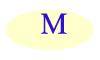

Supplement: Additional file 2 — MetNetGE source code. This is the source code of MetNetGE. Please go to MetNetGE.org to find the latest version, dependency packages and tutorials. [file 1471-2105-11-469-S2.ZIP › MetNetGE-1.0.3/NetworkGE/Sample/basedata/files/FCM/metabolite.png]

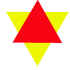

Supplement: Additional file 2 — MetNetGE source code. This is the source code of MetNetGE. Please go to MetNetGE.org to find the latest version, dependency packages and tutorials. [file 1471-2105-11-469-S2.ZIP › MetNetGE-1.0.3/NetworkGE/Sample/basedata/files/FCM/NegaReg.png]

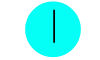

Supplement: Additional file 2 — MetNetGE source code. This is the source code of MetNetGE. Please go to MetNetGE.org to find the latest version, dependency packages and tutorials. [file 1471-2105-11-469-S2.ZIP › MetNetGE-1.0.3/NetworkGE/Sample/basedata/files/FCM/or.png]

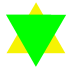

Supplement: Additional file 2 — MetNetGE source code. This is the source code of MetNetGE. Please go to MetNetGE.org to find the latest version, dependency packages and tutorials. [file 1471-2105-11-469-S2.ZIP › MetNetGE-1.0.3/NetworkGE/Sample/basedata/files/FCM/PosiReg.png]

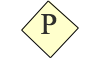

Supplement: Additional file 2 — MetNetGE source code. This is the source code of MetNetGE. Please go to MetNetGE.org to find the latest version, dependency packages and tutorials. [file 1471-2105-11-469-S2.ZIP › MetNetGE-1.0.3/NetworkGE/Sample/basedata/files/FCM/Protein2.png]

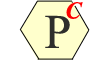

Supplement: Additional file 2 — MetNetGE source code. This is the source code of MetNetGE. Please go to MetNetGE.org to find the latest version, dependency packages and tutorials. [file 1471-2105-11-469-S2.ZIP › MetNetGE-1.0.3/NetworkGE/Sample/basedata/files/FCM/ProteinComplex2.png]

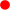

Supplement: Additional file 2 — MetNetGE source code. This is the source code of MetNetGE. Please go to MetNetGE.org to find the latest version, dependency packages and tutorials. [file 1471-2105-11-469-S2.ZIP › MetNetGE-1.0.3/NetworkGE/Sample/basedata/files/FCM/reddot.png]

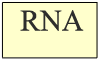

Supplement: Additional file 2 — MetNetGE source code. This is the source code of MetNetGE. Please go to MetNetGE.org to find the latest version, dependency packages and tutorials. [file 1471-2105-11-469-S2.ZIP › MetNetGE-1.0.3/NetworkGE/Sample/basedata/files/FCM/RNA2.png]

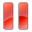

Supplement: Additional file 2 — MetNetGE source code. This is the source code of MetNetGE. Please go to MetNetGE.org to find the latest version, dependency packages and tutorials. [file 1471-2105-11-469-S2.ZIP › MetNetGE-1.0.3/NetworkGE/Sample/basedata/files/QTUI/PauseRed.png]

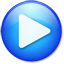

Supplement: Additional file 2 — MetNetGE source code. This is the source code of MetNetGE. Please go to MetNetGE.org to find the latest version, dependency packages and tutorials. [file 1471-2105-11-469-S2.ZIP › MetNetGE-1.0.3/NetworkGE/Sample/basedata/files/QTUI/Play1Hot.png]

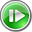

Supplement: Additional file 2 — MetNetGE source code. This is the source code of MetNetGE. Please go to MetNetGE.org to find the latest version, dependency packages and tutorials. [file 1471-2105-11-469-S2.ZIP › MetNetGE-1.0.3/NetworkGE/Sample/basedata/files/QTUI/StepForward.png]

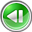

Supplement: Additional file 2 — MetNetGE source code. This is the source code of MetNetGE. Please go to MetNetGE.org to find the latest version, dependency packages and tutorials. [file 1471-2105-11-469-S2.ZIP › MetNetGE-1.0.3/NetworkGE/Sample/basedata/files/QTUI/StepPrevious.png]

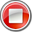

Supplement: Additional file 2 — MetNetGE source code. This is the source code of MetNetGE. Please go to MetNetGE.org to find the latest version, dependency packages and tutorials. [file 1471-2105-11-469-S2.ZIP › MetNetGE-1.0.3/NetworkGE/Sample/basedata/files/QTUI/Stop.png]

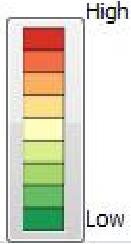

Supplement: Additional file 2 — MetNetGE source code. This is the source code of MetNetGE. Please go to MetNetGE.org to find the latest version, dependency packages and tutorials. [file 1471-2105-11-469-S2.ZIP › MetNetGE-1.0.3/NetworkGE/Sample/basedata-simple/files/Legend-expr.jpg]

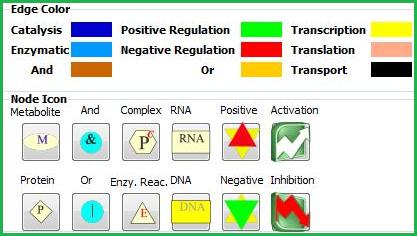

Supplement: Additional file 2 — MetNetGE source code. This is the source code of MetNetGE. Please go to MetNetGE.org to find the latest version, dependency packages and tutorials. [file 1471-2105-11-469-S2.ZIP › MetNetGE-1.0.3/NetworkGE/Sample/basedata-simple/files/Legend.jpg]

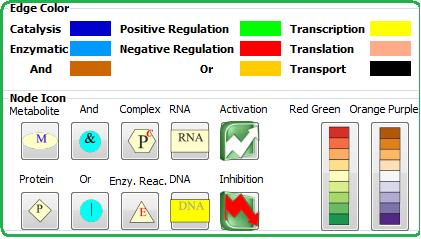

Supplement: Additional file 2 — MetNetGE source code. This is the source code of MetNetGE. Please go to MetNetGE.org to find the latest version, dependency packages and tutorials. [file 1471-2105-11-469-S2.ZIP › MetNetGE-1.0.3/NetworkGE/Sample/basedata-simple/files/Legend2.jpg]

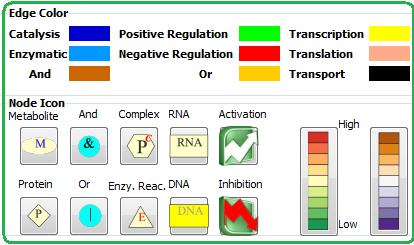

Supplement: Additional file 2 — MetNetGE source code. This is the source code of MetNetGE. Please go to MetNetGE.org to find the latest version, dependency packages and tutorials. [file 1471-2105-11-469-S2.ZIP › MetNetGE-1.0.3/NetworkGE/Sample/basedata-simple/files/Legend3.jpg]

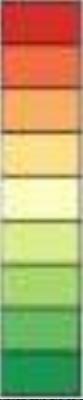

Supplement: Additional file 2 — MetNetGE source code. This is the source code of MetNetGE. Please go to MetNetGE.org to find the latest version, dependency packages and tutorials. [file 1471-2105-11-469-S2.ZIP › MetNetGE-1.0.3/NetworkGE/Sample/basedata-simple/files/RedGreen-large.jpg]

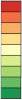

Supplement: Additional file 2 — MetNetGE source code. This is the source code of MetNetGE. Please go to MetNetGE.org to find the latest version, dependency packages and tutorials. [file 1471-2105-11-469-S2.ZIP › MetNetGE-1.0.3/NetworkGE/Sample/basedata-simple/files/RedGreen.jpg]

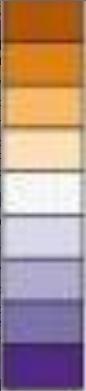

Supplement: Additional file 2 — MetNetGE source code. This is the source code of MetNetGE. Please go to MetNetGE.org to find the latest version, dependency packages and tutorials. [file 1471-2105-11-469-S2.ZIP › MetNetGE-1.0.3/NetworkGE/Sample/basedata-simple/files/YellowPurple-large.jpg]

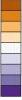

Supplement: Additional file 2 — MetNetGE source code. This is the source code of MetNetGE. Please go to MetNetGE.org to find the latest version, dependency packages and tutorials. [file 1471-2105-11-469-S2.ZIP › MetNetGE-1.0.3/NetworkGE/Sample/basedata-simple/files/YellowPurple.jpg]

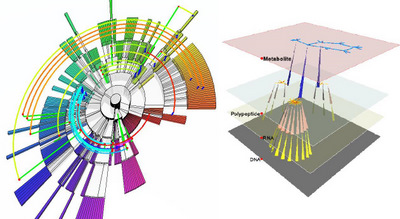

Supplement: Additional file 2 — MetNetGE source code. This is the source code of MetNetGE. Please go to MetNetGE.org to find the latest version, dependency packages and tutorials. [file 1471-2105-11-469-S2.ZIP › MetNetGE-1.0.3/NetworkGE/Sample/basedata-simple/files/ForWiki/logo-big.JPG]

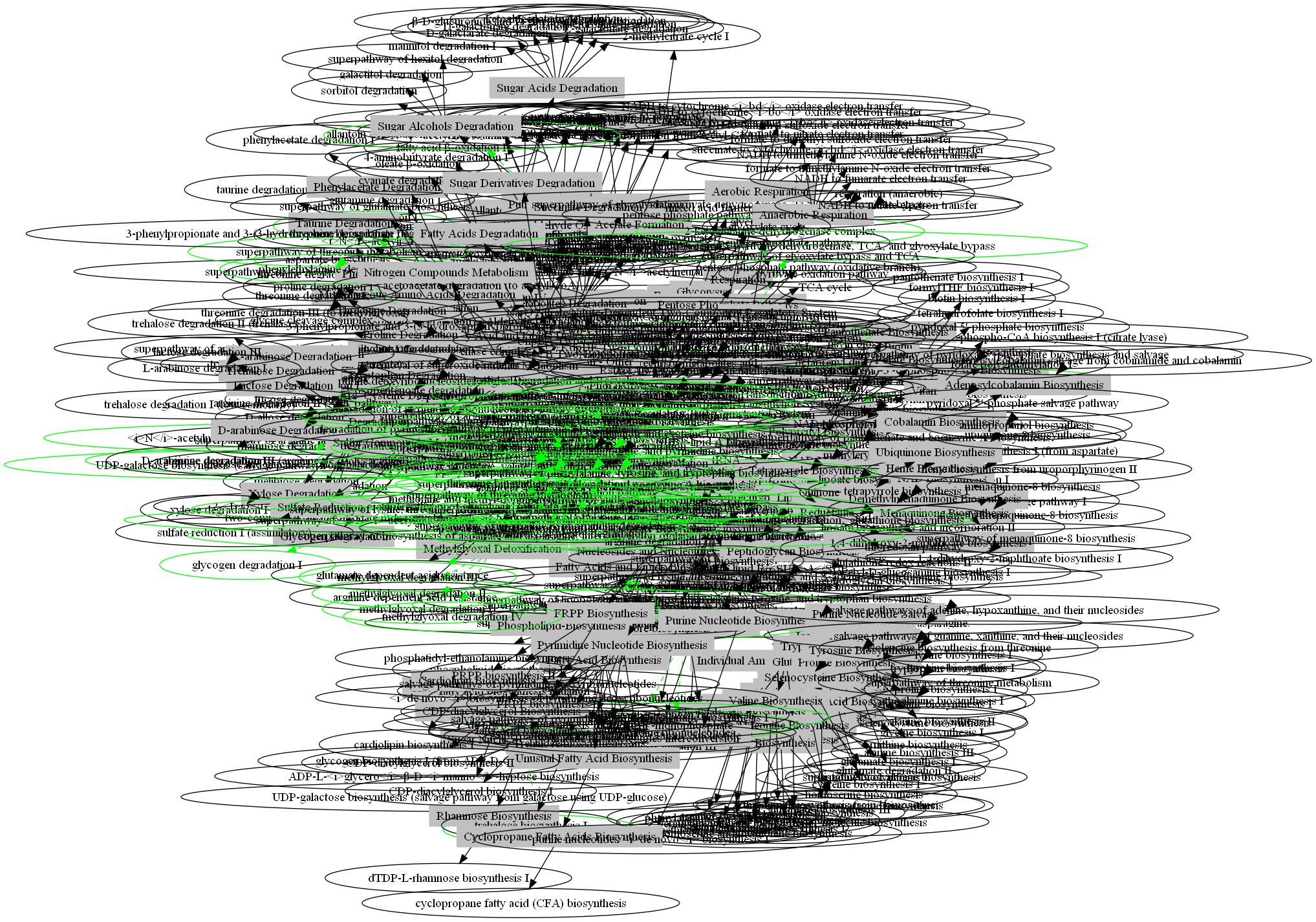

Supplement: Additional file 2 — MetNetGE source code. This is the source code of MetNetGE. Please go to MetNetGE.org to find the latest version, dependency packages and tutorials. [file 1471-2105-11-469-S2.ZIP › MetNetGE-1.0.3/SampleData/ecocyc-all-separate/ecocyc-ontology-hierarchy-neato.jpg]

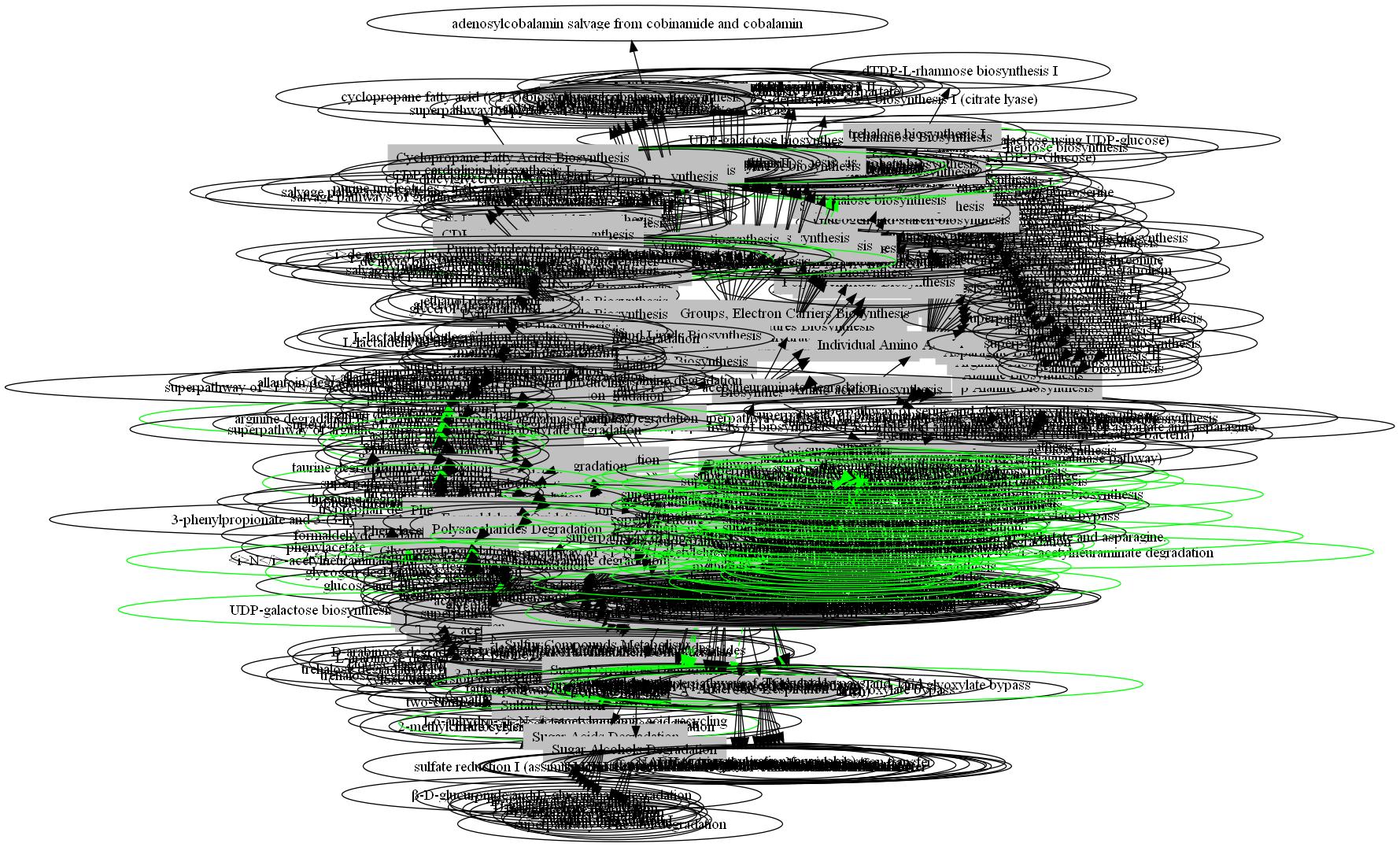

Supplement: Additional file 2 — MetNetGE source code. This is the source code of MetNetGE. Please go to MetNetGE.org to find the latest version, dependency packages and tutorials. [file 1471-2105-11-469-S2.ZIP › MetNetGE-1.0.3/SampleData/ecocyc-all-separate/ecocyc-ontology-hierarchy-twopi.jpg]

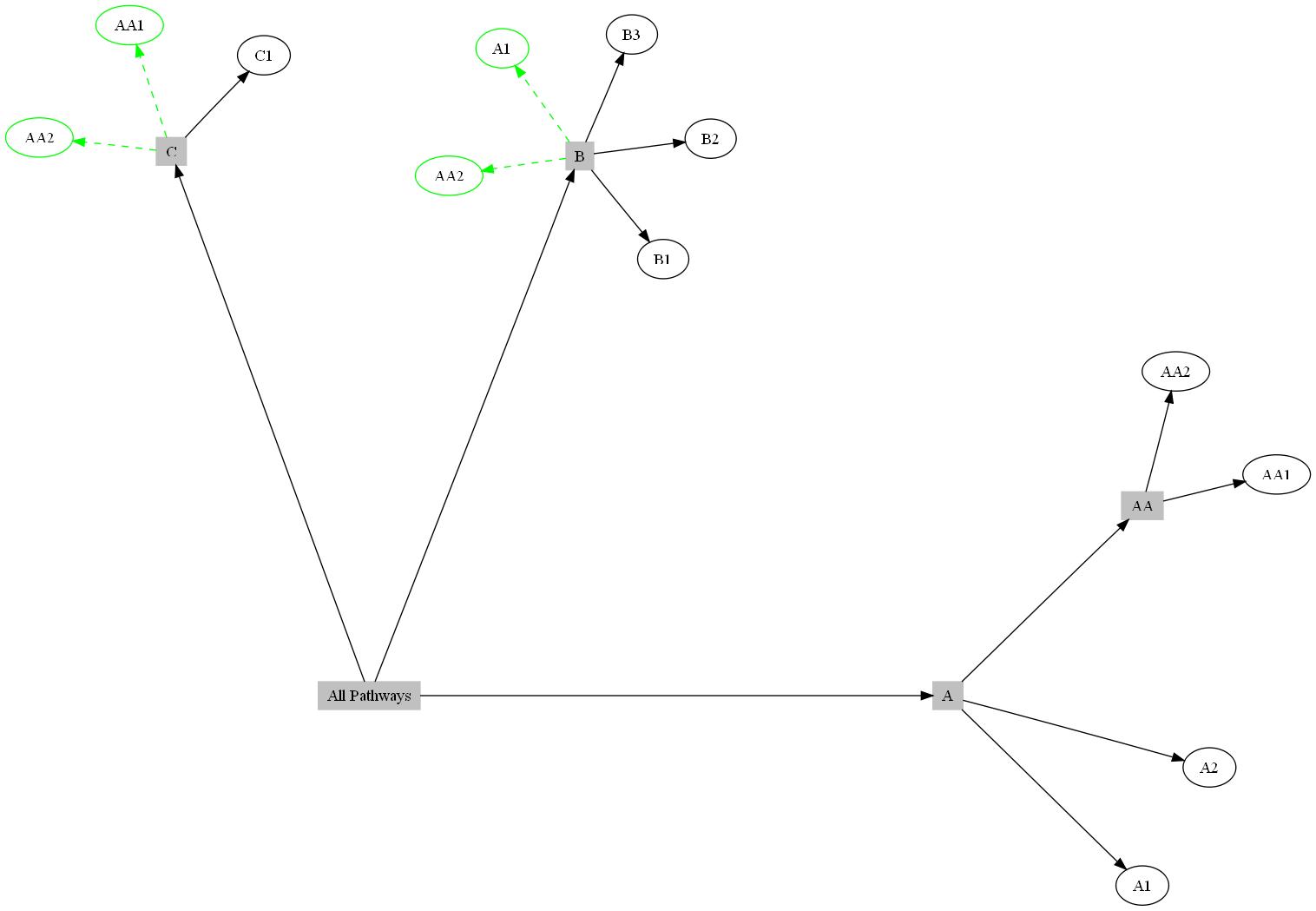

Supplement: Additional file 2 — MetNetGE source code. This is the source code of MetNetGE. Please go to MetNetGE.org to find the latest version, dependency packages and tutorials. [file 1471-2105-11-469-S2.ZIP › MetNetGE-1.0.3/SampleData/ecocyc-all-separate/ecocyc-ontology-test3-hierarchy-circo.jpg]

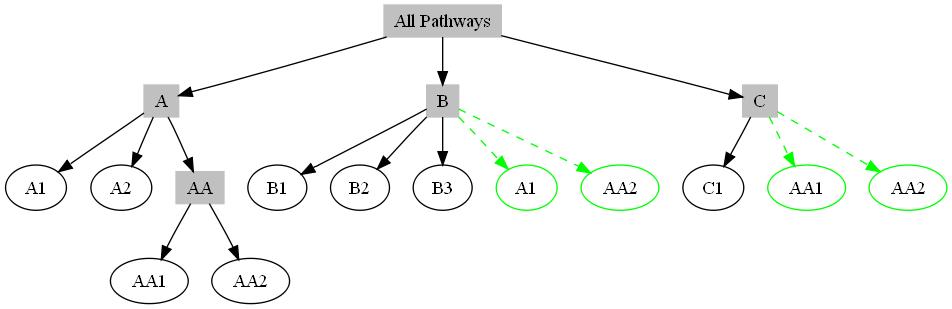

Supplement: Additional file 2 — MetNetGE source code. This is the source code of MetNetGE. Please go to MetNetGE.org to find the latest version, dependency packages and tutorials. [file 1471-2105-11-469-S2.ZIP › MetNetGE-1.0.3/SampleData/ecocyc-all-separate/ecocyc-ontology-test3-hierarchy-dup.jpg]

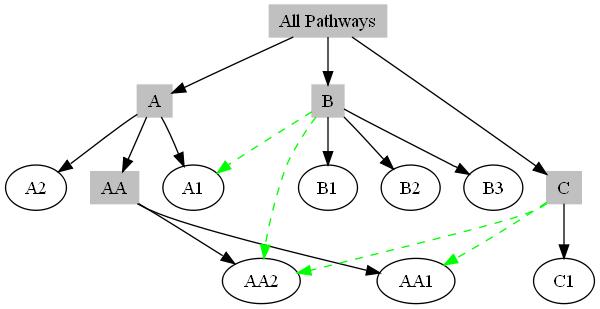

Supplement: Additional file 2 — MetNetGE source code. This is the source code of MetNetGE. Please go to MetNetGE.org to find the latest version, dependency packages and tutorials. [file 1471-2105-11-469-S2.ZIP › MetNetGE-1.0.3/SampleData/ecocyc-all-separate/ecocyc-ontology-test3-hierarchy.jpg]

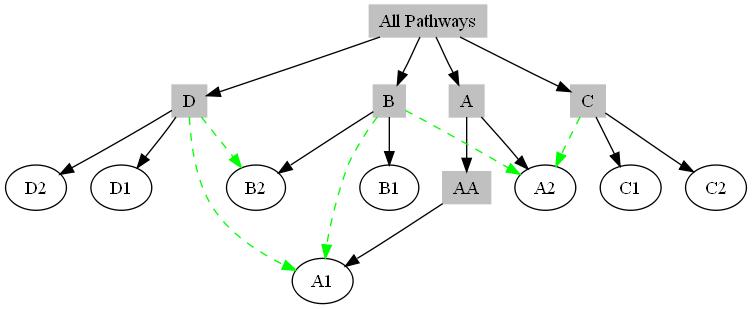

Supplement: Additional file 2 — MetNetGE source code. This is the source code of MetNetGE. Please go to MetNetGE.org to find the latest version, dependency packages and tutorials. [file 1471-2105-11-469-S2.ZIP › MetNetGE-1.0.3/SampleData/ecocyc-all-separate/ecocyc-test-nontree.jpg]

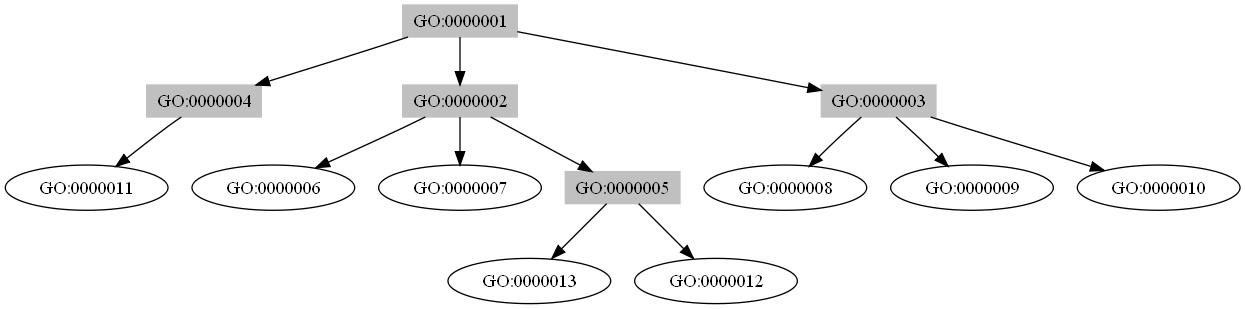

Supplement: Additional file 2 — MetNetGE source code. This is the source code of MetNetGE. Please go to MetNetGE.org to find the latest version, dependency packages and tutorials. [file 1471-2105-11-469-S2.ZIP › MetNetGE-1.0.3/SampleData/GeneOntology/gene_ontology.1_2-sample-hierarchy-dot-id.jpg]

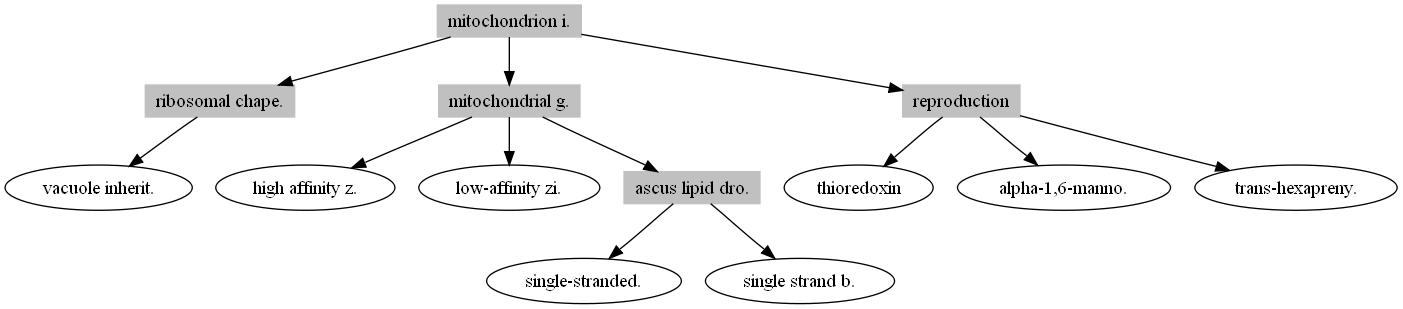

Supplement: Additional file 2 — MetNetGE source code. This is the source code of MetNetGE. Please go to MetNetGE.org to find the latest version, dependency packages and tutorials. [file 1471-2105-11-469-S2.ZIP › MetNetGE-1.0.3/SampleData/GeneOntology/gene_ontology.1_2-sample-hierarchy-dot-sname.jpg]

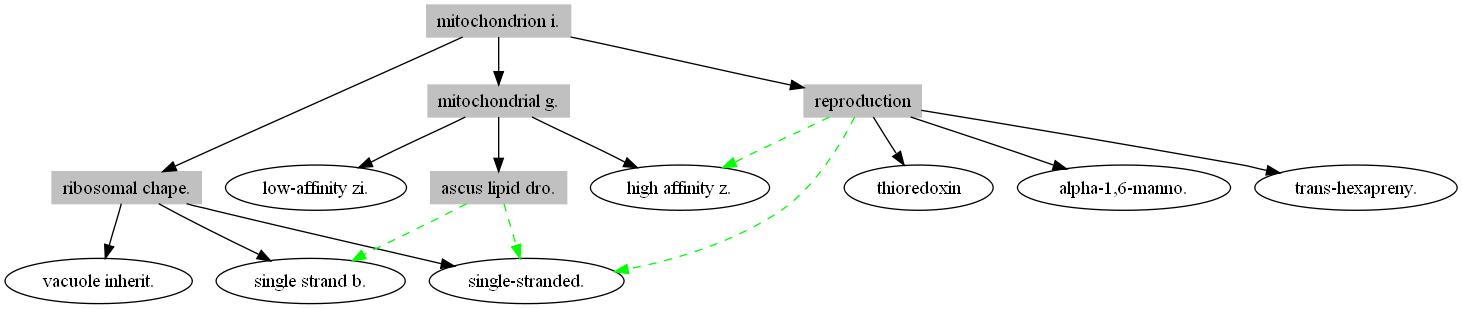

Supplement: Additional file 2 — MetNetGE source code. This is the source code of MetNetGE. Please go to MetNetGE.org to find the latest version, dependency packages and tutorials. [file 1471-2105-11-469-S2.ZIP › MetNetGE-1.0.3/SampleData/GeneOntology/gene_ontology.1_2-sample2-hierarchy-dot-sname.jpg]

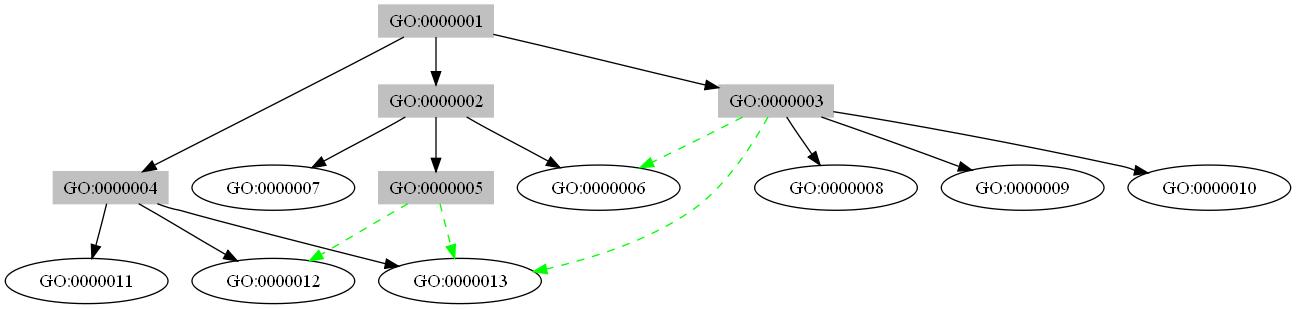

Supplement: Additional file 2 — MetNetGE source code. This is the source code of MetNetGE. Please go to MetNetGE.org to find the latest version, dependency packages and tutorials. [file 1471-2105-11-469-S2.ZIP › MetNetGE-1.0.3/SampleData/GeneOntology/gene_ontology.1_2-sample2-hierarchy-dot.jpg]
