# Supplementary material for: MetNetGE: interactive views of biological networks and ontologies
Source: BMC Bioinformatics. 2010 Sep 17;11:469. doi: 10.1186/1471-2105-11-469 (PMC2946353; doi:10.1186/1471-2105-11-469)
Supplement: Additional file 2 — MetNetGE source code. This is the source code of MetNetGE. Please go to MetNetGE.org to find the latest version, dependency packages and tutorials. [file 1471-2105-11-469-S2.ZIP › MetNetGE-1.0.3/SampleData/LauraDemo/LauraDemoDescription.doc]

Here is some sample E. coli data.

1. We treated the cells with either 4uM DeaNO, 8uM DeaNO or serine hydroxamate (SeOH).
2. The value is the average log ratio from a set of biological and technical replicates, as determined by Markov Chain Monte Carlo analysis. Value is the log(measure/original).
3. DeaNO is a nitric oxide (NO) donor and serine hydroxamate is a serine analog that induces serine starvation.

Statistics after log

mean:-0.000566628583182, min:-0.933905, max:1.44709, sum:-7.1327206051

Statistics before log, which makes more sense

mean:1.035, min:0.1164, max:27.9956, sum: 13034.92

**Intrested Pathways**

superpathway of fatty acid biosynthesis

http://ecocyc.org/ECOLI/new-image?object=PWY0-881

Subpathways:

| FullName |  | ShortName | ID |
| --- | --- | --- | --- |
| fatty acid biosynthesis initiation I |  | PWY-4381 | A1 |
| fatty acid biosynthesis initiation II |  | PWY-5966 | A2 |
| fatty acid biosynthesis initiation III |  | PWY-5965 | B1 |
| fatty acid elongation -- saturated |  | FASYN-ELONG-PWY | B2 |
|  |  |  |  |
| fatty acid biosynthesis - initial steps I |  | FASYN-INITIAL-PWY | C2 |
| alanine degradation I |  | ALADEG-PWY | C1 |
| acetyl-CoA biosynthesis (from pyruvate) |  | PYRUVDEHYD-PWY | D1 |
| N-acetylglucosamine degradation |  | GLUAMCAT-PWY | D2 |
|  |  |  |  |

**Mapping Rules**

| Visual Value | Mapped Value | Description |
| --- | --- | --- |
| Region Color | GeneExpr Abs Value | 1. absolute experiment value of a gene at a condition. 2. The average of (1) for all genes in the group. |
|  | GeneExpr Abs Diff | 1. The absolute difference between value at one condition and previous condition. 2. The average of (1) for all genes in the group. |
| Region Inner-Outer Height | Range Value | 1. Experiment value of a gene at a condition. (some offset) 2. The range of (1) for all genes in the group. |
|  | Range (Coeff of Variation) | 1. Variation among all experiments for a single gene.  2. The range of (1) for genes in all the group. |

**Toy dataset hierarchy:**
